# Supplementary material for: Eliminating HIV-1 Packaging Sequences from Lentiviral Vector Proviruses Enhances Safety and Expedites Gene Transfer for Gene Therapy
Source: Mol Ther. 2017 May 24;25(8):1790–804. doi: 10.1016/j.ymthe.2017.04.028 (PMC5542766; doi:10.1016/j.ymthe.2017.04.028)
Supplement: Document S1. Figures S1–S7 [file mmc1.pdf]

YMTHE, Volume *25*

Supplemental Information

Eliminating HIV-1 Packaging Sequences from Lentiviral Vector Proviruses Enhances Safety and Expedites Gene Transfer for Gene Therapy

Conrad A. Vink, John R. Counsell, Dany P. Perocheau, Rajvinder Karda, Suzanne M.K. Buckley, Martijn H. Brugman, Melanie Galla, Axel Schambach, Tristan R. McKay, Simon N. Waddington, and Steven J. Howe

Supplementary Materials

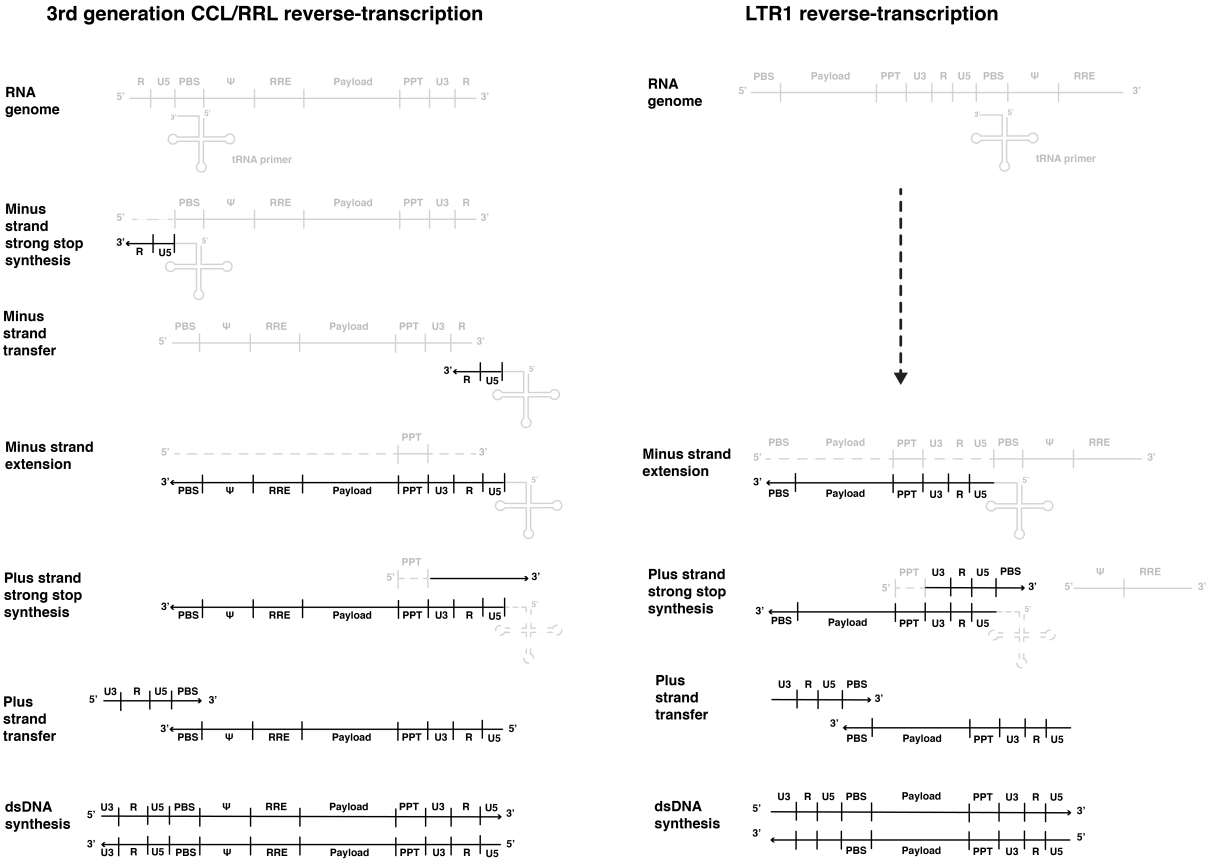

Supplementary Figure S1. LTR1 reverse-transcription requires fewer intermediate steps than a standard lentiviral vector. For all images, grey lines represent RNA, black lines represent the DNA product of reverse-transcription, and dashed grey lines represent RNA degraded by RNAse H. **Left panel.** Conventional lentiviral reverse-transcription can be broken-down in intermediate steps. A tRNA primer binds to the HIV-1 primer-binding site (PBS) on vector RNA and initiates minus strong-stop DNA synthesis, producing single-stranded DNA (ssDNA) complementary to the R-U5 portion of the HIV-1 LTR. Vector RNA is concurrently degraded by RNAse H, thus leaving R-U5 ssDNA free to base pair with the complementary R domain at the 3’ end of the vector RNA. Reverse transcriptase proceeds to complete synthesis of minus strand DNA, with PBS at the 5’terminus. Plus strand synthesis is then primed from the polypurine tract (PPT), reading through the 3’LTR (U3-R) and the tRNA, which is complementary to PBS. A single-stranded PBS is now present at both ends, which

permits the second strand-transfer event. Reverse transcriptase then proceeds to complete plus strand synthesis to produce a double-stranded provirus. **Right panel.** LTR1 reverse-transcription operates with fewer intermediate steps than standard lentiviruses. LTR1 vectors lack a 5’LTR, so reverse-transcription is primed on an internal PBS downstream of a solitary HIV-1 LTR (U3-R-U5 domains). This means that reverse-transcriptase can complete minus strand synthesis without performing a strand transfer event and skip straight to minus strand extension. Like conventional reverse-transcription, plus strand synthesis is primed from the PPT, reading through the full-length 3’LTR and tRNA, which is complementary to the PBS. Reading through the tRNA primer displaces the untranscribed RNA at the extreme 3’ end. This process displaces the internal PBS and excludes the HIV-1 packaging sequences from reverse-transcription. As per conventional reverse-transcription, a single-stranded PBS is now present at both DNA termini, which permits the second strand-transfer event and synthesis of a double-stranded DNA provirus. With Ψ-RRE sequences situated downstream of all reverse-transcription events, these sequences are not incorporated into the final LTR1 provirus.

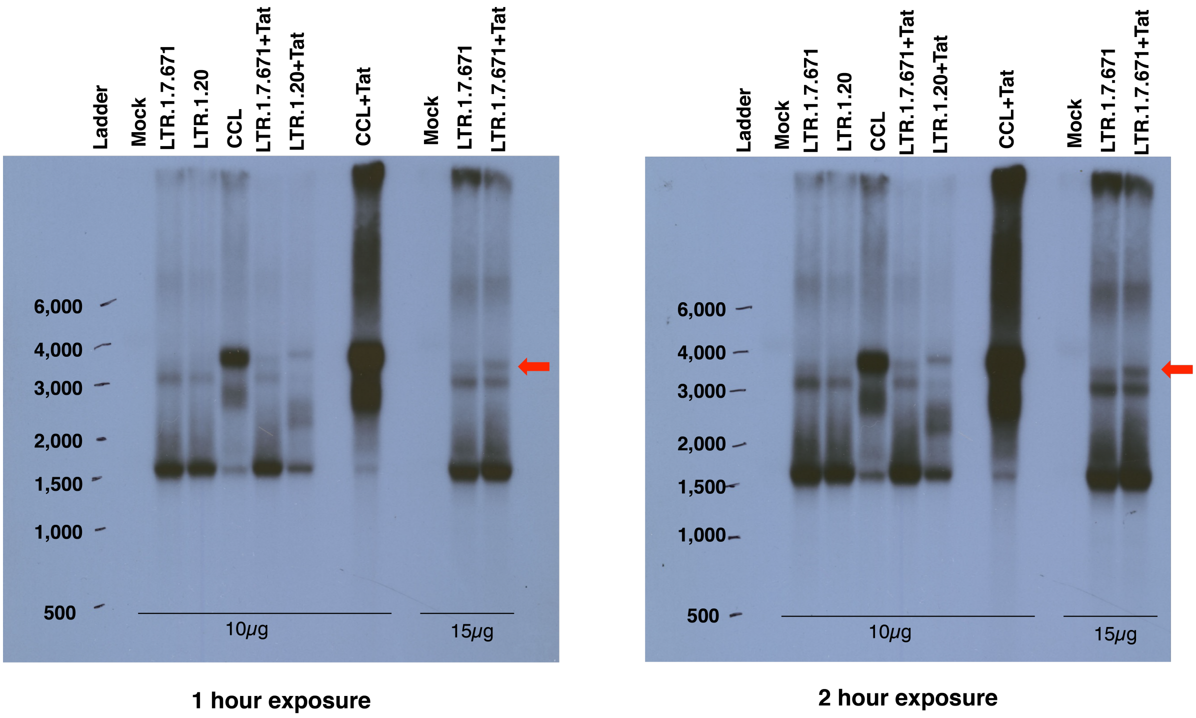

Supplementary Figure S2. Northern blot analysis of LTR1 RNA products. HEK293T cells were transfected with LTR1.7.671-GEW, LTR1.20-GEW or CCL-GEW plasmids. Vectors were produced with or without pcDNA3.Tat during production. RNA was extracted from each treated cell population and processed for northern blot, where RNA was probed for EGFP. EGFP-probed phosphorescence was exposed for 1 hour (left panel) or 2 hours (right panel) at -80°C. Samples were loaded with mass of 10μg, with LTR1.7.671-GEW also loaded at 15μg. Red arrow points to the LTR1.7.671-GEW band assumed to be full-length vector genome transcript. The mock RNA sample was extracted from untreated HEK 293T cells.

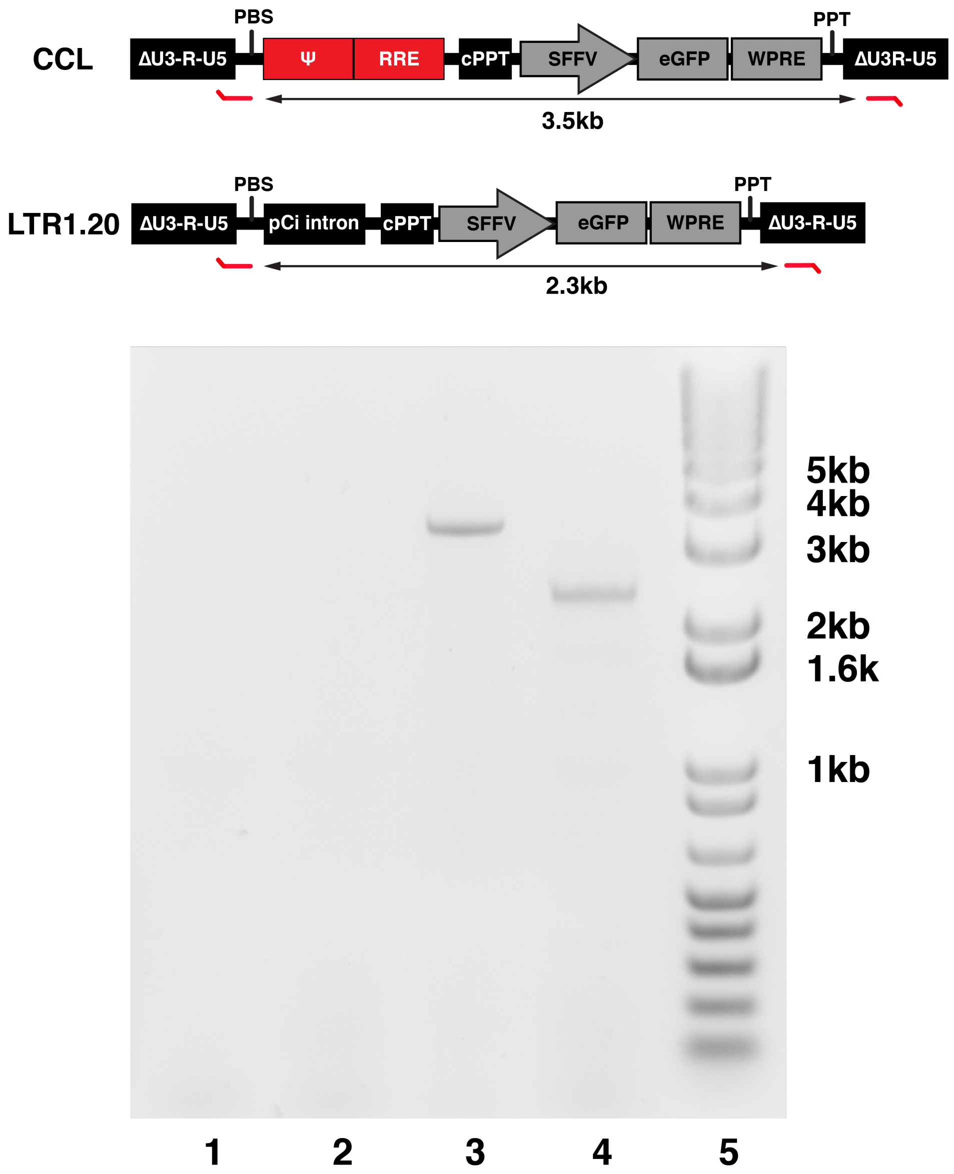

Supplementary Figure S3. PCR amplification of LTR1.20-SFFV-eGFP provirus. Genomic DNA of HT1080 cells transduced with either CCL-SFFV-eGFP (lane 3) or LTR1.20-SFFV-eGFP (lane 4) was amplified using primers directed against the flanking lentiviral LTRs. Water (lane 1) and untransduced HT1080 genomic DNA (lane 2) were devoid of any non-specific amplification. PCR products were analysed alongside the Invitrogen 10kb+ DNA ladder (lane 5), which showed that CCL (3.5kb) and LTR1.20 (2.3kb) samples produced amplicons of the expected sizes. The primer binding sites relative to each provirus have been shown above the gel image.

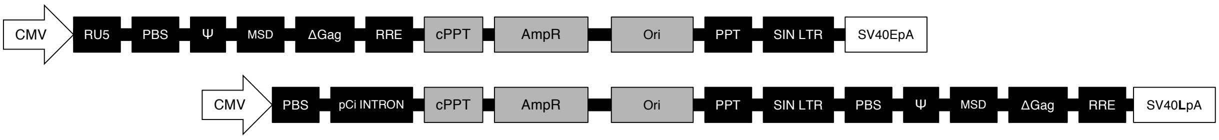

Supplementary Figure S4. Plasmid rescue vector genomes. The pBR322 selection marker, containing an ampicillin resistance gene and bacterial origin of replication, was cloned into the vector transgene region of pCCL (top) or pLTR.20 (bottom). AmpR – ampicillin resistance gene for bacterial selection; Ori – pBR322 plasmid origin of replication for propagation in bacterial cells.

CTTCCCCCTTACCTGACAGCTGTTATTAGATTACGGCCGAATGTCCAATCTTTAACATCATGATGTAGG
GCTCTGTGGTTAACCCATTGCCAAATGTTTTATTATTTCCATAGGTTTAGTGGTCCCAACTTCGGATCTC
ATGTGAGGAAGGTCAAAATGACAGCTTGAAAATATTCCTAACCCGGGGATTGCAGTGCCCATAAGAAAT
GATTGCCAGTGGGGAGAAAGCCCATTTGCTGATTCCACATCTTGTGCTAGCTTTTACACAAATCATCTC
ACAGA**AGAAT**TGGAAGGGCTAATTCACTCCCAACGAAGACAAGATCTGCTTTTTGCTTGTACTGGGTCT
CTCTGGTTAGACCAGATCTGAGCCTGGGAGCTCTCTGGCTAACTAGGGAACCCACTGCTTAAGCCTCA
ATAAAGCTTGCCTTGAGTGCTTCAAGTAGTGTGTGCCCGTCTGTTGTGTGACTCTGGTAACTAGAGATC
CCTCAGACCCTTTTAGTCAGTGTGGAAAATCTCTAGCAGTGGCGCCCGAACAGGGACTGCTAGCCTGG
GCAGGTGTCCACTCCCAGTTCGCTAGCGTTAACTTTTAAAAGAAAAGGGGGGATTGGGGGGTACAGTG
CAGGGGAAAGAATAGTAGACATAATAGCAACAGACATACAAACTAAAGAATTACAAAAACAAATTACAA
AAATTCAAAATTTTATCGATCACGAGACTAGCCTCGAGCCAGGTGGCACTTTTCGGGGAAATGTGCGC
GGAACCCCTATTTGTTTATTTTTCTAAATACATTCAAATATGTATCCGCTCATGAGACAATAACCCTGATA
AATGCTTCAATAATATTGAAAAAGGAAGAGTATGAGTATTCAACATTTCCGTGTCGCCCTTATTCCCTTT
TTTGCGGCATTTTGCCTTCCTGTTTTTGCTCACCCAGAAACGCTGGTGAAAGTAAAAGATGCTGAAGAT
CAGTTGGGTGCACGAGTGGGTTACATCGAACTGGATCTCAACAGCGGTAAGATCCTTGAGAGTTTTCG
CCCCGAAGAACGTTTTCCAATGATGAGCACTTTTAAAGTTCTGCTATGTGGCGCGGTATTATCCCGTAT
TGACGCCGGGCAAGAGCAACTCGGTCGCCGCATACACTATTCTCAGAATGACTTGGTTGAGTACTCAC
CAGTCACAGAAAAGCATCTTACGGATGGCATGACAGTAAGAGAATTATGCAGTGCTGCCATAACCATG
AGTGATAACACTGCGGCCAACTTACTTCTGACAACGATCGGAGGACCGAAGGAGCTAACCGCTTTTTT
GCACAACATGGGGGATCATGTAACTCGCCTTGATCGTTGGGAACCGGAGCTGAATGAAGCCATACCAA
ACGACGAGCGTGACACCACGATGCCTGTAGCAATGGCAACAACGTTGCGCAAACTATTAACTGGCGAA
CTACTTACTCTAGCTTCCCGGCAACAATTAATAGACTGGATGGAGGCGGATAAAGTTGCAGGACCACTT
CTGCGCTCGGCCCTTCCGGCTGGCTGGTTTATTGCTGATAAATCTGGAGCCGGTGAGCGTGGGTCTC
GCGGTATCATTGCAGCACTGGGGCCAGATGGTAAGCCCTCCCGTATCGTAGTTATCTACACGACGGGG
AGTCAGGCAACTATGGATGAACGAAATAGACAGATCGCTGAGATAGGTGCCTCACTGATTAAGCATTG
GTAACTGTCAGACCAAGTTTACTCATATATACTTTAGATTGATTTAAAACTTCATTTTTAATTTAAAAGGA
TCTAGGTGAAGATCCTTTTTGATAATCTCATGACCAAAATCCCTTAACGTGAGTTTTCGTTCCACTGAGC
GTCAGACCCCGTAGAAAAGATCAAAGGATCTTCTTGAGATCCTTTTTTTCTGCGCGTAATCTGCTGCTT
GCAAACAAAAAAACCACCGCTACCAGCGGTGGTTTGTTTGCCGGATCAAGAGCTACCAACTCTTTTTCC
GAAGGTAACTGGCTTCAGCAGAGCGCAGATACCAAATACTGTCCTTCTAGTGTAGCCGTAGTTAGGCC
ACCACTTCAAGAACTCTGTAGCACCGCCTACATACCTCGCTCTGCTAATCCTGTTACCAGTGGCTGCTG
CCAGTGGCGATAAGTCGTGTCTTACCGGGTTGGACTCAAGACGATAGTTACCGGATAAGGCGCAGCG
GTCGGGCTGAACGGGGGGTTCGTGCACACAGCCCAGCTTGGAGCGAACGACCTACACCGAACTGAGA
TACCTACAGCGTGAGCTATGAGAAAGCGCCACGCTTCCCGAAGGGAGAAAGGCGGACAGGTATCCGG
TAAGCGGCAGGGTCGGAACAGGAGAGCGCACGAGGGAGCTTCCAGGGGGAAACGCCTGGTATCTTTA
TAGTCCTGTCGGGTTTCGCCACCTCTGACTTGAGCGTCGATTTTTGTGATGCTCGTCAGGGGGGCGGA
GCCTATGGAAAAACGCCAGCAACGCGGCCTTTTTACGGTTCCTGGCCTTTTGCTGGCCTTTTGCTCACA
TGTGAATTCGAGCTCGGTACCTTTAAGACCAATGACTTACAAGGCAGCTGTAGATCTTAGCCACTTTTT
AAAAGAAAAGGGGGGACTGGAAGGGCTAATTCACTCCCAACGAAGACAAGATCTGCTTTTTGCTTGTA
CTGGGTCTCTCTGGTTAGACCAGATCTGAGCCTGGGAGCTCTCTGGCTAACTAGGGAACCCACTGCTT
AAGCCTCAATAAAGCTTGCCTTGAGTGCTTCAAGTAGTGTGTGCCCGTCTGTTGTGTGACTCTGGTAAC
TAGAGATCCCTCAGACCCTTTTAGTCAGTGTGGAAAATCTCTAGCA**AGAAT**CCTTACACTCTCTGATGC
AGGAGACAATATCCCCAATCACAGGTGAGGGTGCTGACATTAGAGAAGCAAGCCATTCAGCAACAGCT
TAGGGGAAGAGCTCTCAAATGTAGTGTTTTCATACTCCAAAGTTCAGAGGAAGTGTTTCTTTCCCTTAAA
ATGGCAGCAGTTTTCAATTCTGATGTTATAGCTCAGAAGTGGGGACAGAGAGGGATAGTAGAAGGCTG
CCAAATGACATTAAATGAATTTTTTTCATGACAAAGTAATTCCTCAGAATCAGTTTTTTTCCCATTACCTA
ATTGAAGTATCATTATATTCTCATGTTTAATATGTTATTTAGCATATCACCAAGCAGATTGAAAAGGCCG
AAAATGAGCAAAAGAATGCACATATCACTCACTCATTGCCTGTCTTTGCTCTCTCCCTCATGAAGACATA
GAAGAGGAGGCTAGGGGATGCCCCCTCCTCTTCCCTCAGACAGGGGATTCTCACTGACAGAAATATGT
AATTCTTCACATTGCTTTAAAAATGTTCTTCTTCATGTGTATTTATGTGTTTTCAGCTTACTACTCTTATAC
CACCTGCTGTTCAAACAAAAAATCCCACCAAGCCAAGCAGAATTGATGAATATCCATTTACTTTTATTTT
AAAGGGTGCCTCAACTCTTTCAAATCCTAAAATCTTTACCGTATTAATTTGTTAAGGCTTCCTTTAGTAA
GTCAGGGTAAACCCTAA

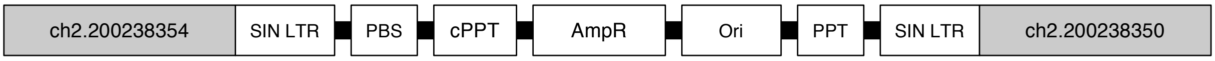

Supplementary Figure S5. Sequence composition of representative plasmid rescue clone. The sequencing data for the rescued LTR1.20 provirus confirms the expected internal provirus structure that would be produced following pCi intron splicing in producer cells (non-shaded text) and shows the repeated dinucleotide pairs at either end of the integrated provirus (bold text) resulting from genomic integration. The shaded text is homologous to human chromosome 2, flanking the repeated dinucleotides at position 200238350-200238354. The map represents the contents and structure of the provirus, devoid of HIV-1 packaging sequences.

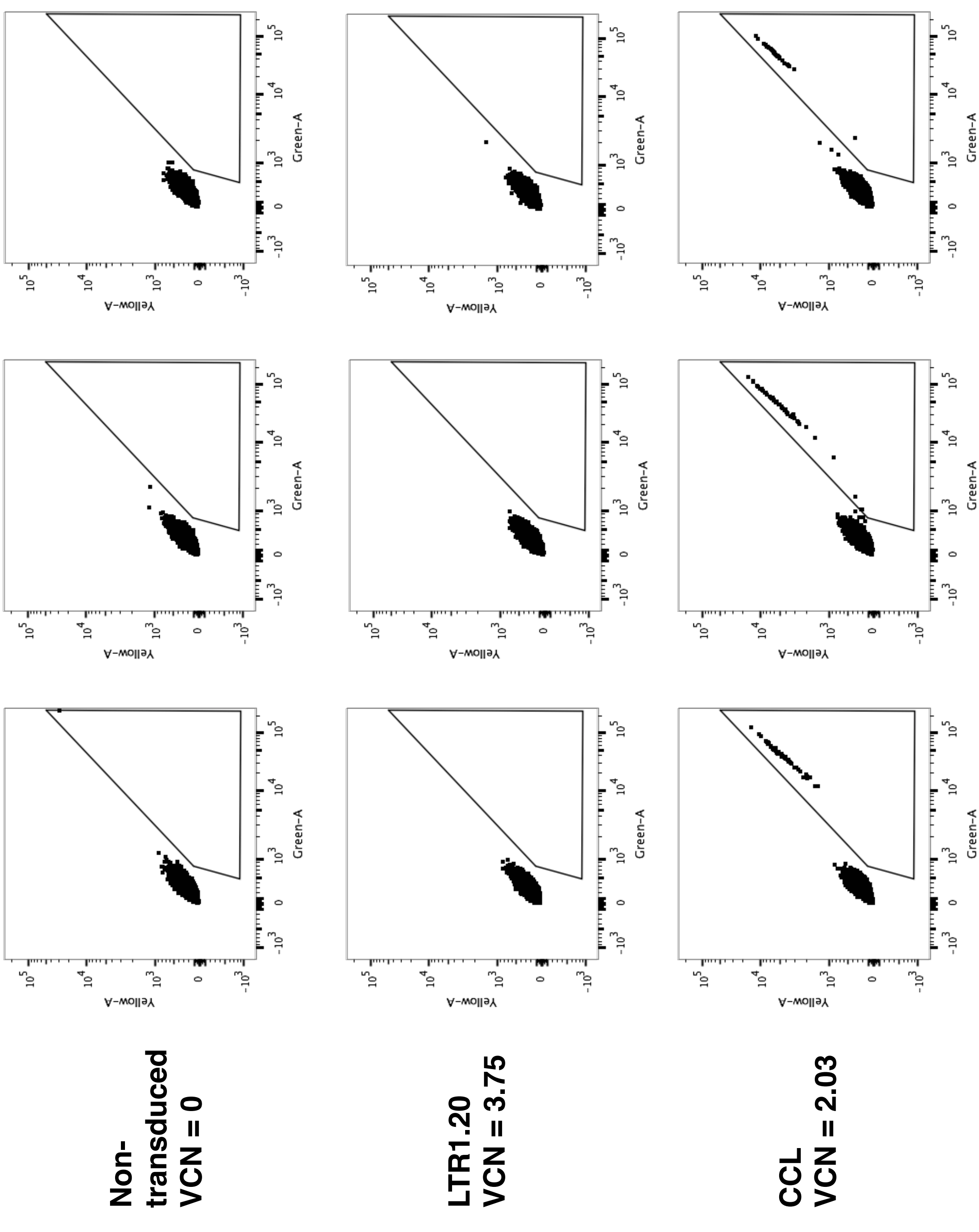

<u>Supplementary Figure S6. Flow cytometry dot plots for remobilisation assay samples.</u> HEK 293T cells transducd with mock remobilised vectors were analysed by flow cytometry for GFP positivity. Vector remobilised by a CCL sample of VCN 2.03 gave clear remobilised GFP vector titres, whilst LTR1.20 showed resistance to remobilisation even a higher VCN dose of 3.75.

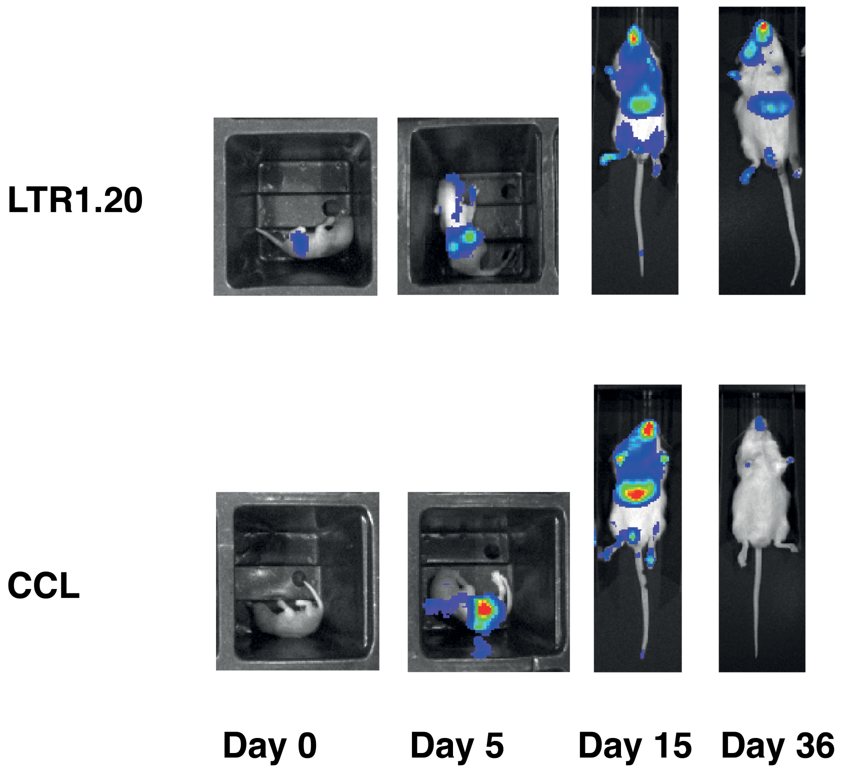

Supplementary Figure S7. Representative bioluminescent images of SFFV-Luc-T2A-eGFP-transduced animals. Outbred CD1 mice received titre-matched intravenous doses of either LTR1.20-SFFV-Luc-T2A-eGFP or CCL-SFFV-Luc-T2A-eGFP at postnatal day 1. Mice were imaged continually throughout the following 36 days to track vector expression *in vivo*. Here, we show representative images taken at 20mins (day 0), 5 days, 15 days and 36 days post-administration.
