# Supplementary material for: Eliminating HIV-1 Packaging Sequences from Lentiviral Vector Proviruses Enhances Safety and Expedites Gene Transfer for Gene Therapy
Source: Mol Ther. 2017 May 24;25(8):1790–804. doi: 10.1016/j.ymthe.2017.04.028 (PMC5542766; doi:10.1016/j.ymthe.2017.04.028)
Supplement: Document S2. Article plus Supplemental Information [file mmc2.pdf]

Molecular Therapy
Original Article

Eliminating HIV-1 Packaging Sequences from Lentiviral Vector Proviruses Enhances Safety and Expedites Gene Transfer for Gene Therapy

Conrad A. Vink,[1,7] John R. Counsell,[1,2,7] Dany P. Perocheau,[2] Rajvinder Karda,[2] Suzanne M.K. Buckley,[2] Martijn H. Brugman,[4] Melanie Galla,[5] Axel Schambach,[5] Tristan R. McKay,[6] Simon N. Waddington,[2,3] and Steven J. Howe[1,2]

[1]Molecular and Cellular Immunology, UCL Great Ormond Street Institute of Child Health, 30 Guilford Street, London WC1N 1EH, UK; [2]Gene Transfer Technology Group, Institute for Women's Health, University College London, 86-96 Chenies Mews, London WC1E 6HX, UK; [3]MRC Antiviral Gene Therapy Research Unit, Faculty of Health Sciences, University of the Witswatersrand, Johannesburg 2000, South Africa; [4]Department of Immunohematology and Blood Transfusion, Leiden University Medical Center, 2300 RC Leiden, the Netherlands; [5]Institute of Experimental Hematology, Hannover Medical School, 30625 Hannover, Germany; [6]School of Healthcare Science, John Dalton Building, Manchester Metropolitan University, Chester Street, Manchester M15 6BH, UK

Lentiviral vector genomic RNA requires sequences that partially overlap wild-type HIV-1 *gag* and *env* genes for packaging into vector particles. These HIV-1 packaging sequences constitute 19.6% of the wild-type HIV-1 genome and contain functional *cis* elements that potentially compromise clinical safety. Here, we describe the development of a novel lentiviral vector (LTR1) with a unique genomic structure designed to prevent transfer of HIV-1 packaging sequences to patient cells, thus reducing the total HIV-1 content to just 4.8% of the wild-type genome. This has been achieved by reconfiguring the vector to mediate reverse-transcription with a single strand transfer, instead of the usual two, and in which HIV-1 packaging sequences are not copied. We show that LTR1 vectors offer improved safety in their resistance to remobilization in HIV-1 particles and reduced frequency of splicing into human genes. Following intravenous luciferase vector administration to neonatal mice, LTR1 sustained a higher level of liver transgene expression than an equivalent dose of a standard lentivirus. LTR1 vectors produce reverse-transcription products earlier and start to express transgenes significantly quicker than standard lentiviruses after transduction. Finally, we show that LTR1 is an effective lentiviral gene therapy vector as demonstrated by correction of a mouse hemophilia B model.

INTRODUCTION

The *Retroviridae* family of viruses was first investigated as vectors for mammalian gene transfer over 30 years ago, and this technology continues to develop.[1,2] The current generations of lentiviral vectors are based on HIV type 1 (HIV-1),[3–6] in which the vector RNA must contain a significant portion of the wild-type HIV-1 genome to enable successful packaging into virus particles.

The HIV-1 elements that must be present in lentiviral vectors include the RNA packaging signal (Ψ), the major splice donor, and the Rev-response element (RRE), which are implicated in vector RNA processing and packaging into viral particles.[7–10] During viral particle assembly, the nucleocapsid protein recognizes and binds to Ψ to ensure that RNA is encapsidated by the budding virion.[11,12] The RRE interacts with the HIV-1 Rev protein to stabilize transcripts and promote RNA export from the nucleus.[13–18]

Following transduction of target cells, the RNA genome in a standard lentiviral vector is reverse-transcribed to form a double-stranded DNA (dsDNA) provirus. The HIV-1 long terminal repeats (LTRs) mark the boundaries of the reverse-transcribed template; thus, Ψ and RRE are always incorporated into standard lentiviral vector proviruses and integrated into the host cell genome as they are situated between the LTRs.

The persistence of HIV-1-derived elements in target cells presents a risk for clinical translation of lentiviral technology due to potential interactions between virus and patient genomes. The Ψ and RRE portions of lentiviral vector DNA contain functional *cis* elements, such as the major splice donor, which can disrupt cellular processes in patient cells. For example, it has been shown that integrated lentiviral proviruses can upregulate the human growth hormone receptor due to interactions between the HIV-1 major splice donor and splice acceptors in the human growth hormone receptor gene.[19] Additionally, it has been observed that splice acceptors in lentiviral vector packaging sequences can splice with patient genes to create aberrant fusion transcripts.[20] This was recently observed in a gene therapy clinical trial for β-thalassemia in which splicing between the patient HMGA2 gene

Received 25 December 2016; accepted 28 April 2017;
http://dx.doi.org/10.1016/j.ymthe.2017.04.028.

[7]These authors contributed equally to this work.

Correspondence: John R. Counsell, Molecular and Cellular Immunology, UCL Great Ormond Street Institute of Child Health, 30 Guilford Street, London WC1N 1EH, UK.
E-mail: j.counsell@ucl.ac.uk

 © 2017 The Author(s).
This is an open access article under the CC BY license (http://creativecommons.org/licenses/by/4.0/).

and an integrated lentiviral provirus caused dysregulation of HMGA2 transcription and a large clonal expansion of a transduced cell.[21] The presence of the Ψ packaging element in vector proviruses presents further problems by enabling remobilization of proviral transcripts in HIV-1-infected cells.[22–24] Provirus remobilization in HIV-1- infected patients has been cited as a primary safety concern in the clinical use of lentiviral vectors.[25,26] If standard lentiviruses were used in HIV-positive patients, there is a possible risk of lentiviral vector proviruses being remobilized by HIV factors supplied in *trans* and for potential recombination events with wild-type HIV-1 genomes. Furthermore, any transgene cassette remobilized in HIV-1 particles could be transferred to any newly infected individuals.

Previous attempts to remove Ψ and RRE from lentiviral vector proviruses have been largely unsuccessful. Cui et al.[27] attempted to further truncate the Ψ and RRE sequences in the lentiviral vector genome in efforts to minimize the transfer of HIV-1 DNA. This resulted in aberrant splicing of the vector genome in HEK293T producer cells and functional vector propagation was found to be dependent on the use of an unconventional TE671 rhabdomyosarcoma producer cell line. Delviks et al.[28] attempted to exploit homologous recombination events that occur during viral reverse-transcription to "skip" the vector packaging signal during provirus synthesis, although this technique operated with limited efficiency as skipping did not always occur. More recently, Cre-loxP mediated deletion of the Ψ and RRE sequences was used to markedly reduce the prevalence of these sequences in transduced cells, although again, the level of efficiency was limited by the efficiency of recombination and Cre expression in target cells may be undesirable or unfeasible in clinical applications.[29] These investigations exemplify the technical challenges that complicate the removal of HIV-1 packaging sequences from clinical-grade vectors.

Here, we describe the development and initial application of a novel lentiviral vector, LTR1, in which the HIV-1 packaging sequences have been relocated to avoid their transfer into target cell nuclei. In LTR1 vector RNA, the HIV-1 Ψ and RRE packaging sequences are located downstream of a single self-inactivating LTR (sin.LTR).[5] This positioning ensures that the necessary HIV-1 structures are present for efficient RNA packaging and processing in producer cells, but absent from the delivered provirus due to their exclusion from reverse-transcription. As a result, LTR1 proviruses contain just 441 bp of the wild-type HIV-1 genome, which is limited to the vector LTRs, primer binding site, and polypurine tracts. We have iteratively optimized the structure of LTR1 to achieve high titers for in vivo gene therapy applications, and we have investigated LTR1 gene transfer both in vitro and in vivo to compare this system to standard lentiviral technology.

RESULTS

Optimization of LTR1 Genome to Achieve Vector Titers Sufficient for Gene Therapy

The premise of LTR1 technology is to generate an RNA genome that mimics the first strand that is synthesized during the initial stages of reverse-transcription in target cells (known as the "minus strand"). This is achieved in LTR1 vectors by removing the vector 5′LTR, thus leaving the HIV-1 primer binding site at the extreme 5′ terminus of the vector RNA. An additional primer binding site is situated immediately downstream of a solitary self-inactivating LTR (ΔU3 SIN),[5] followed by the necessary packaging sequences. The location of primer binding site immediately downstream of the LTR ensures the necessary proximity of the upstream primer activation signal, which is present within the LTR U5 region, thus guaranteeing efficient initiation of reverse-transcription.[30,31] The LTR1 design means that reverse-transcription moves from two essential strand transfer events to just one, thus shortening the virus life-cycle. A schematic for the expected mechanism of LTR1 reverse-transcription is detailed in Figure S1, and the expected RNA and DNA products are displayed in Figure 1.

During iterative LTR1 development, vector transduction efficiency was determined by flow cytometry, by delivering GFP and the woodchuck-hepatitis virus post-transcriptional regulatory element (WPRE), driven by either the human phosphoglycerate kinase (PGK) promoter, or human glyceraldehyde-3-phosphate dehydrogenase (GAPDH) promoter. The first iteration of LTR1 was produced by editing a third generation pRRL-PGK-EGFP-WPRE lentiviral plasmid (RRL-PEW),[4] for which the primer binding site and Ψ-RRE packaging sequences were moved to a new location between the 3′sin.LTR and early simian virus 40 polyadenylation sequence (SV40 polyA) (Figure 2A). This vector, termed LTR1.0-PGK-EGFP-WPRE (LTR.1.0-PEW), gave an infectious, EGFP titer of 1.2×10^5 transducing units per milliliter (TU/mL) following concentration by ultracentrifugation, almost three orders of magnitude lower than the standard RRL-PEW vector (3.4×10^8 TU/mL; data not shown), as determined by flow cytometric quantification of EGFP expression in transduced 293T cells (Figure 2B).

To increase LTR1 titers to a level suitable for gene therapy applications, the LTR1 genomic structure was progressively optimized with the goal of obtaining yields similar to the original third generation lentivirus from which LTR1 was developed (Figure 2A). Two major bottlenecks to producing high titer vector were believed to be insufficient nuclear export of vector RNA and undesirable transcription termination in the 3′sin.LTR. The majority of the optimization steps were designed to tackle these restrictions. The initial modifications that improved vector titers included exchanging the SV40 early polyA for the SV40 late polyA for improved polyadenylation (LTR1.5-PEW), use of the cytomegalovirus (CMV) promoter to increase expression of vector genomic RNA in producer cells (LTR1.7.671-GEW), and insertion of a small chimeric intron into the vector 5′ UTR with the intention of increasing nuclear export (LTR1.20-GEW).[32] The GAPDH promoter was introduced during development of LTR1.7.671-GEW, as we observed patchy EGFP fluorescence in producer cells when using the PGK promoter (data not shown).

Full-length LTR1 and CCL vector RNA would be expected to produce a 4 kb transcript, when all packaging elements are incorporated.

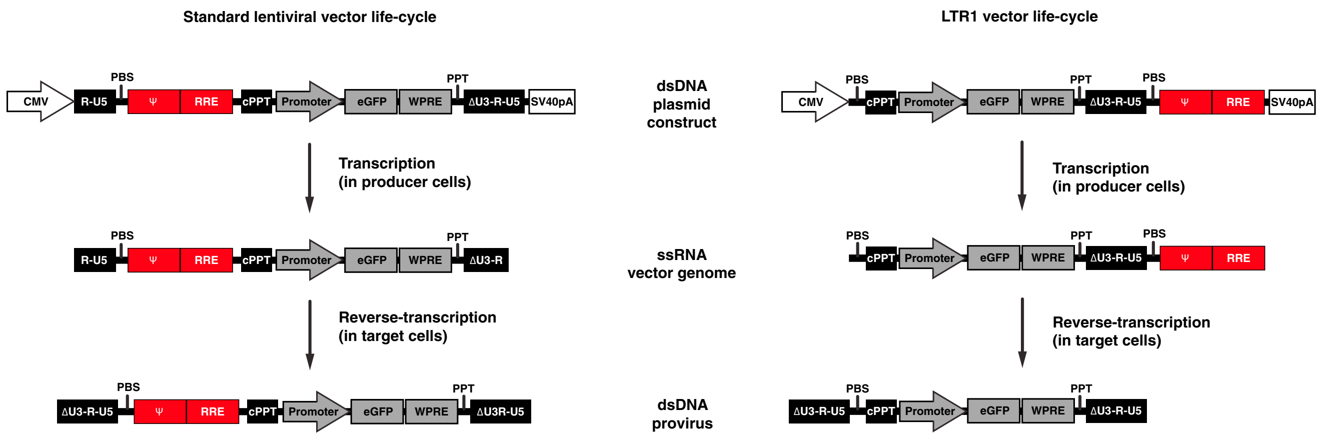

Figure 1. The Structure of LTR1 Gives Rise to RNA and DNA Products that Are Distinct from CCL

CCL and LTR1 plasmid genomes (top row) are both driven by a CMV promoter to produce ssRNA vector genomic transcripts in producer cells (second row). CCL genomic transcripts are flanked by the HIV-1 R region at their extreme termini, whereas LTR1 is rearranged, so that the transcript contains a primer binding site (PBS) at its extreme 5′ terminus and an additional primer binding site adjacent to a solitary self-inactivating LTR, with HIV-1 packaging sequences (Ψ-RRE is highlighted in red boxes) situated downstream of both primer binding site sequences. This means that reverse-transcription initiates upstream of Ψ-RRE, and these sequences are not converted into double-stranded DNA. The result is that LTR1 products are devoid of Ψ-RRE and are thus smaller than CCL products.

However, northern blotting of vector RNA derived from LTR1.7.671 and LTR1.20 producer cells showed a strong band at approximately 1.7 kb when third generation packaging plasmids (without Tat expression) were used, while the wild-type CCL-GEW sample gave the expected 4 kb band at a greater frequency (Figure S2). Co-transfection with pcDNA3.Tat during vector production was able to rescue the 4 kb band in northern blotted LTR1.7.671-GEW and LTR1.20-GEW genomic RNA, indicating that efficient processing of LTR1 vector RNA during vector production is relatively dependent on the presence of HIV-1 Tat in producer cells. For this reason, Tat was included during the production of all LTR1 and CCL vectors for the remainder of the experiments.

The close proximity of the CMV enhancer to the internal GAPDH promoter in pLTR1.20-GEW and pLTR1.7.671-GEW may have strengthened the activity of this internal GAPDH promoter in producer cells, thus resulting in some vector transcripts being initiated from the GAPDH promoter itself (rather than CMV) and consequentially missing important upstream elements (primer binding site and cPPT). To combat potential CMV enhancement of the internal GAPDH promoter, we introduced larger introns in the 5′ UTR to effectively add space between the two promoters. Insertion of a truncated β-globin intron (LTR1.25-GEW) or an elongation factor 1 α intron (LTR1.27-GEW) was successful in increasing the functional titer ($p = 0.004$ by Kruskal-Wallis comparison of all vector titers) (Figure 2B). At this stage, LTR1.27 titers can be produced with efficiency equivalent to approximately 35% of a standard lentiviral vector (pCCL) titer.

LTR1 Proviruses Are Devoid of HIV-1 Packaging Sequences

The absence of HIV-1 packaging sequences from the delivered provirus is fundamental to the benefits of LTR1 technology. To confirm that the packaging signal is not copied during reverse-transcription, two methods were used to sequence LTR1-derived DNA proviruses in transduced cell lines.

Initially, a PCR was carried out using genomic DNA harvested from HT1080 cells transduced with an LTR1.20 vector containing EGFP expressed by the spleen focus-forming virus promoter (SFFV) (LTR1.20-SFFV-EGFP). The PCR was designed to amplify the region spanning from the 5′LTR-primer binding site junction up to the R component of the 3′LTR. Resolution of the PCR product by agarose gel electrophoresis showed that LTR1.20 products were approximately 2.4 kb, which matched the expected size of products lacking HIV-1 packaging sequences (Figure S3). This confirmed that LTR1.20-SFFV-EGFP products are indeed smaller than those derived from CCL-SFFV-EGFP, with an estimated provirus size difference of 1.3 kb. The sequence composition of the PCR products was confirmed by Sanger sequencing, which matched the expected provirus structures (data not shown).

A "plasmid rescue" method[33] was also implemented to confirm the structure of LTR1 product by enabling recovery and direct sequencing of integrated proviruses from transduced HEK293T cells. In this process, the pBR322 bacterial origin of replication and selection marker were relocated to the transgene region of the pLTR1.20 plasmid to allow propagation of the provirus products in competent bacteria, following provirus recovery from transduced mammalian cells (Figure S4). Sequencing of LTR1.20-rescue proviruses confirmed that the expected provirus structure, with Ψ and RRE removed, was present in transduced cells, and these products had successfully integrated into the cell genome with expected dinucleotide repeats (Figure S5).

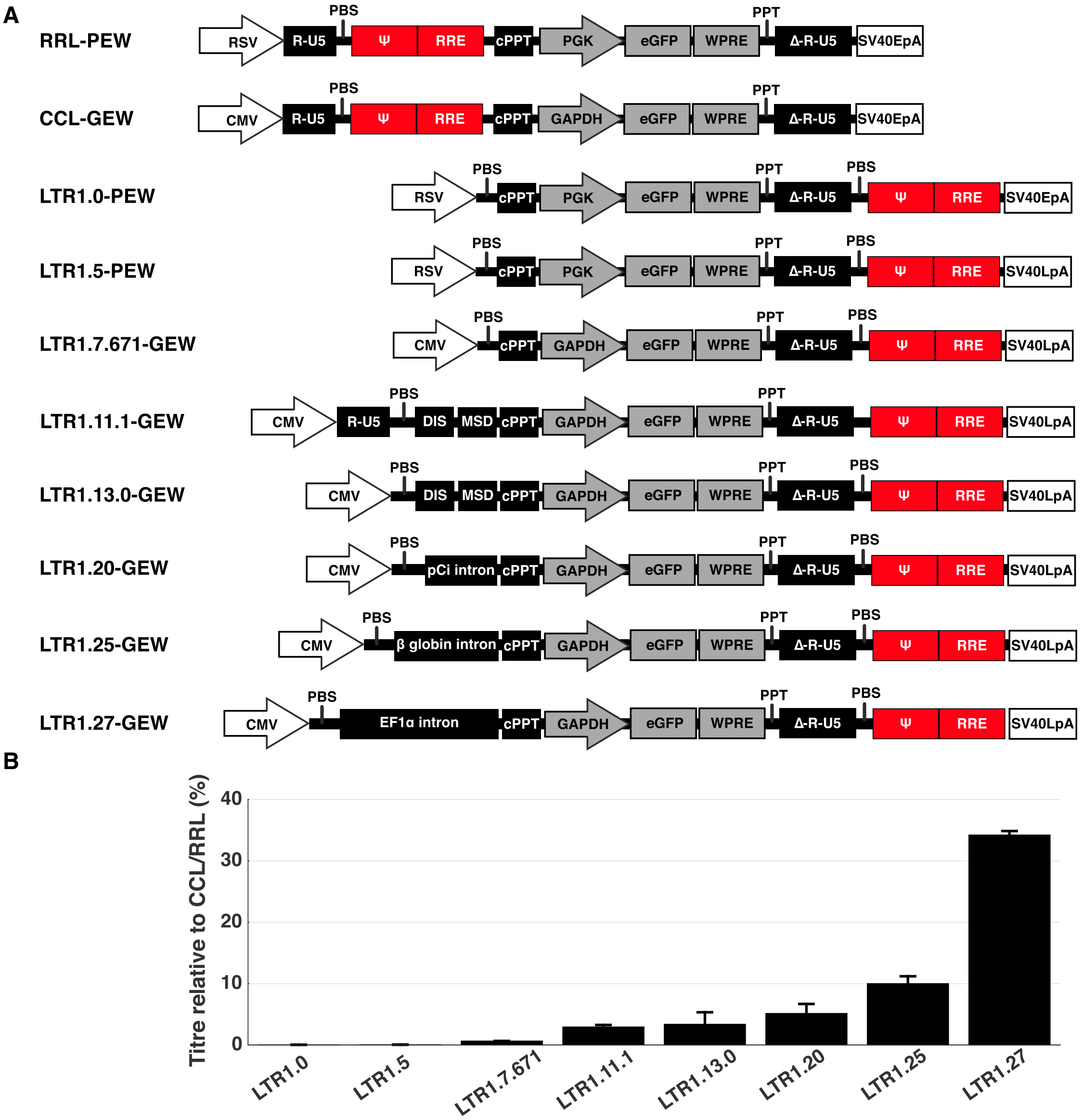

Figure 2. Key Stages of the LTR1 Development Process

(A) LTR1 genomes were developed based on the pRRL-PGK-EGFP (pRRL-PEW) or pCCL-GAPDH-EGFP (pCCL-GEW) third generation lentiviral plasmids. pLTR1.0 was made by transferring the pRRL packaging sequences downstream of the 3′ self-inactivating LTR. pLTR1.5 introduced the late SV40 polyA site (SV40LpA) in place of the early SV40 polyA site (SV40EpA). pLTR1.7.671 replaced the viral RSV promoter with the CMV promoter. pLTR1.11.1 was designed to investigate the influence of minus-strand exchange, with the 5′LTR components returned to the genome. This was directly compared with pLTR1.13.0, which was similar to pLTR1.11.1, but lacked a 5′LTR and had a primer binding site returned to the 3′ end, to remove minus-strand exchange. pLTR1.20 introduced a 133 bp chimeric intron to mimic the effects of HIV-1 splice sites. pLTR1.25 and pLTR1.27 were designed to further improve titers by examining the influence of the 476 bp human β-globin intron and the 939 bp human elongation factor 1 alpha (EF1α) intron upstream of the GAPDH promoter. RSV, Rous sarcoma virus promoter; RU5, R and U5 components of HIV-1 LTR; primer binding site, HIV-1 primer binding site; Ψ, HIV-1 packaging signal; MSD, HIV-1 major splice donor; ΔGag, truncated and inactive HIV-1 gag gene; RRE, HIV-1; cPPT, central polypurine tract; PGK, human phosphoglycerate kinase promoter; WPRE, woodchuck-hepatitis virus post-transcriptional regulatory element; PPT, polypurine tract; SIN LTR, self-inactivating 3′LTR; DIS, HIV-1 dimer initiation sequence; GAPDH, human glyceraldehyde-3-phosphate dehydrogenase promoter; pCi intron, chimera between introns from human β-globin and immunoglobulin heavy chain genes; β-globin intron, internally truncated intron from human β-globin; EF1α intron, intron upstream of human EF1α start codon. (B) Progressive titer increases through LTR1 development process. Stepwise optimization of the LTR1 backbone gradually increased the functional titers from 0.04% of standard third generation levels to the current level of 35% ($p = 0.004$ by Kruskal-Wallis comparison of all titers). All titers are based on functional EGFP output, and LTR1 titrations were performed in parallel with a wild-type third generation vector. LTR1 iterations driven by the RSV viral promoter and PGK internal promoter (LTR1.0 and LTR1.5) were compared to pRRL-PEW titers, while all subsequent LTR1 titers were compared to pCCL-GEW. The bars represent mean values with the error bars showing SEM.

Table 1. Characterization of LTR1 and CCL Vector Composition by Various Titration Methods

Vector	RNA Titer	Particle Titer	Provirus Titer	EGFP Titer	Reverse Transcription Efficiency (%)	Expression Efficiency (%)	Packaging Efficiency (%)
CCL	1.35×10^{11} ssRNAvg/mL	1.68×10^{11} lp/mL	2.23×10^{10} dsDNAvg/mL	1.62×10^{10} TU/mL	33.04	72.80	80.21
LTR1.20	2.24×10^{11} ssRNAvg/mL	2.60×10^{11} lp/mL	1.84×10^{8} dsDNAvg/mL	1.09×10^{8} TU/mL	0.16	58.96	86.32

ssRNAvg/mL, ssRNA vector genomes per mL; lp/mL, lentiviral particles per mL; dsDNAvg/mL, dsDNA vector genome proviruses yielded per mL; TU/mL, EGFP-forming TU/mL.

LTR1 Transduction Characteristics Compared to Third Generation CCL In Vitro

In vitro experiments were carried out to further characterize LTR1 vectors and compare them to a third generation lentiviral vector. For these investigations, we used vectors containing an SFFV-driven bicistronic luciferase-EGFP reporter gene in which luciferase and EGFP are separated by a T2A cleavage peptide (SFFV-Luc-T2A-EGFP).[34] HT1080 cells were transduced with a range of LTR1.20-SFFV-Luc-T2A-EGFP or CCL-SFFV-Luc-T2A-EGFP doses. At 2 weeks after transduction, EGFP expression was analyzed by flow cytometry and vector copy numbers (VCNs) were quantified by qPCR titration of the genomic DNA. Lentiviral titers were determined by p24 ELISA (lp/mL), qRT-PCR of packaged single-stranded RNA genomes (ssRNAvg/mL), qPCR analysis of integrated dsDNA vector genome proviruses (dsDNAvg/mL), and flow cytometric quantification of EGFP-positive cells (TU/mL) (Table 1).

The data in Table 1 show that, while LTR1.20-GEW particle and RNA genome titers are similar to CCL, the provirus titer and EGFP titer appear to be restricted. We further analyzed these parameters by calculating the approximate efficiency of key steps in the viral life-cycle. The relative packaging efficiency was calculated by expressing the RNA titer as a percentage of the particle titer, while the transgene expression efficiency was based on the EGFP titer as a percentage of the provirus titer. The efficiency of LTR1 packaging (86.3%) and transgene expression (59.0%) was similar to CCL (80.2% and 72.8%). Reverse-transcription efficiency was calculated by expressing the provirus titer as a percentage of the RNA genome titer. This value showed that LTR1.20 (0.16%) was less efficient than CCL (33.04%) at converting its RNA genome into a stable provirus.

The similarities and differences between LTR1.20 and CCL vector parameters are shown in more detail in Figure 3. Plotting the integrated VCN versus the mean fluorescence intensity (MFI) of EGFP expression (Figure 3A) shows that transgene expression efficiency per LTR1.20 provirus matches the level derived from the CCL vector. However, when plotting the p24 and RNA genome doses versus the percentage of GFP-positive cells (Figures 3B and 3C), it becomes apparent that LTR1.20 requires a greater total particle number and RNA dose than CCL to achieve an equivalent effect, underlining the potential inefficiency during transduction.

LTR1 Proviruses Are Resistant to Remobilization in the Presence of HIV-1 Packaging Components Provided in *trans*

Despite self-inactivating lentiviruses lacking an active promoter in the 5′LTR,[5] it has been reported that lentivirus-transduced cells can still produce full-length RNA genomes that are remobilized in new virions when host cells are actively producing HIV-1 components.[22,23] Possible remobilization of gene therapy vectors in patients infected with HIV presents significant safety and regulatory concerns.[25,26] We hypothesized that the absence of HIV-1 packaging sequences from LTR1 proviruses would confer resistance to re-packaging of any full-length RNA in target cells where HIV-1-based packaging components were provided in *trans*.

To investigate this, we transduced HEK293T cells with a 2-fold dilution series of LTR1.20-SFFV-EGFP or CCL-SFFV-EGFP and maintained the populations in culture for 11 days. Genomic DNA was harvested from each transduced population for VCN quantification, and the remaining cells were plated out and transfected with lentiviral packaging plasmids to create a pool of mock-HIV-1-infected cells. Vector supernatants were subsequently harvested and purified prior to addition to HT1080 cells, which were analyzed at 10 days post-transduction to quantify any expression from functional vector particles with the ability to deliver stably integrated remobilized viral genomes (Figure 4).

LTR1.20 samples were completely devoid of any remobilized proviruses, revealed by the lack of EGFP expression at all VCN doses. Third generation lentivirus (CCL) samples were positive for EGFP expression, with remobilized titers calculated in the range of 5.9×10^2–1.5×10^4 TU/mL, where area under the curve (AUC) increased in correlation with the starting VCN ($p = 0.0004$ by t test) (Figure 4A).

Three example flow cytometry dot plots show clear EGFP-positive colonies produced by a CCL-derived vector (Figure S6) remobilized from a HEK293T population possessing a CCL VCN of 2.03 vector genomes per cell. LTR1.20 was not remobilized from HEK293T cells containing 3.75 genomes per cell, with plots indistinguishable from a non-transduced negative controls.

The Risk of Generating Vector Host-Fusion Transcripts Is Reduced with LTR1 Technology

Transcripts derived from lentiviral vector proviruses have been shown to fuse with neighboring gene transcripts in patient cells

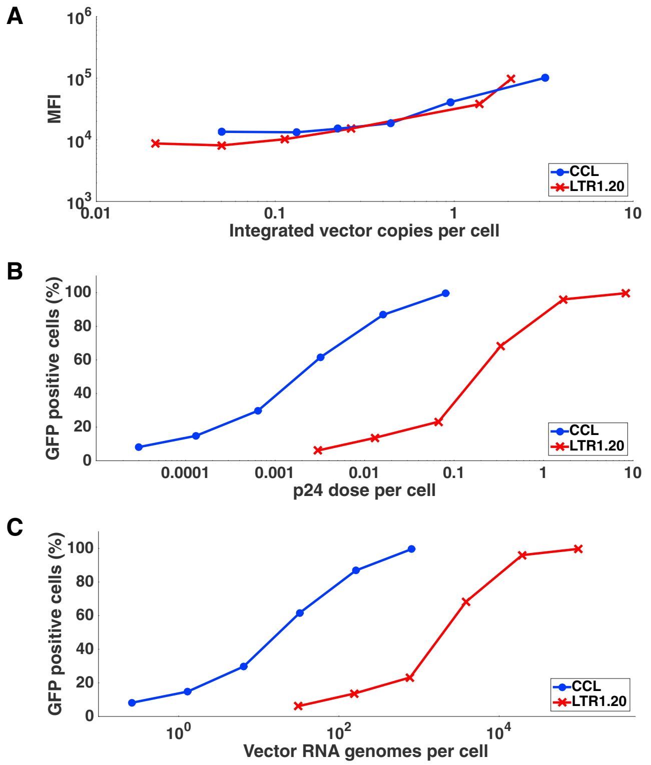

Figure 3. Characterization of LTR1 Vector Titers In Vitro

(A) Plotting the integrated vector copy number versus the mean fluorescence intensity (MFI) of EGFP expression in HT1080 cells 2 weeks after transduction shows that expression intensity from transgenes delivered by LTR1.20-SFFV-Luc-T2A-EGFP (red lines, cross markers) or a third generation CCL vector (blue lines, circular markers) are equivalent per integrated provirus. (B) When comparing the percentage of GFP positive cells versus the vector dose according to gag p24 mass, it becomes apparent that LTR1.20 vectors require a higher particle dose to match the CCL level of efficacy in vitro. (C) Further to this, when plotting the level of GFP positive cells versus the total vector dose in terms of RNA genomes, we see that a greater number of LTR1 genomes are required to match the CCL level of transduction.

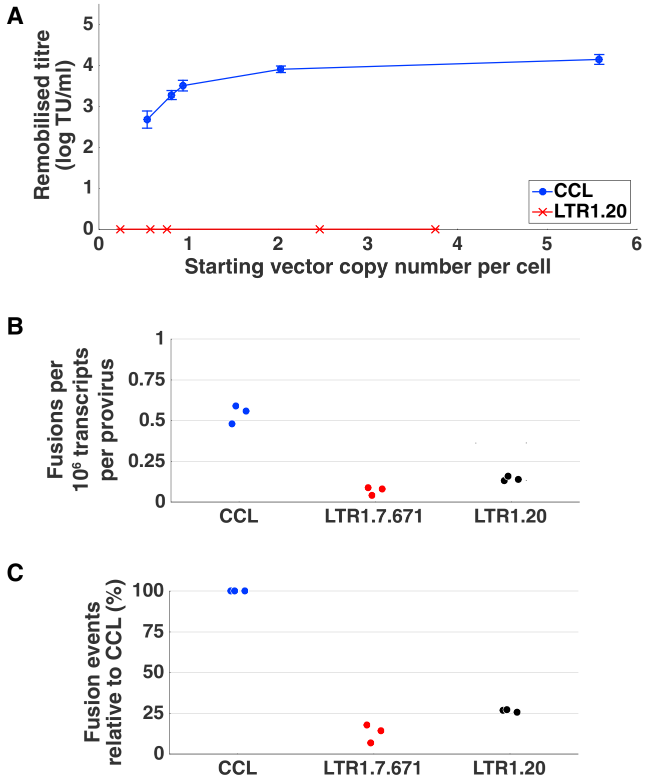

Figure 4. Analyzing the Safety Parameters of CCL and LTR1 Vector Proviruses

(A) HEK293T cells transduced with CCL-SFFV-EGFP vector proviruses produce a remobilized titer that increases in correlation with the number of proviral copies per cell. LTR1.20-SFFV-EGFP proviruses do not produce any detectable remobilized genomes at any dose (n = 3 vector titrations for each VCN dose; data are expressed as mean transducing units per milliliter (TU/mL) ± SEM; (p = 0.0004 following t test comparison of area under the curves). (B) Chimeric vector-human fusion transcripts occur less frequently in LTR1 samples compared to standard lentivirus samples. The transcriptomic profiling of HEK293T cells transduced with either CCL-GEW (n = 3), LTR1.7.671-GEW (n = 3), or LTR1.20-GEW (n = 3) was investigated to assess the influence of Ψ-RRE removal from LTR1 proviruses. The number of fusion transcripts detected in CCL samples per 10^6 human genomic transcripts per integrated provirus was significantly greater than those of LTR1.7.671 and LTR1.20 (p = 0.027 by Kruskal-Wallis test). (C) Expressing the data values relative to the CCL fusion transcript frequency shows that LTR1.7.671 (12.97 ± 5.64% of CCL) and LTR1.20 (26.5 ± 0.86% of CCL) generate chimeric transcripts at a significantly lower rate than CCL (p = 0.024 by Kruskal-Wallis test).

through splicing interactions between HIV-1 splice sites and the human genome.[19–21] This constitutes a safety risk for clinical gene therapy. We hypothesized that the absence of a HIV-1 major splice donor and Ψ-RRE splice acceptors from LTR1 vector proviruses would influence the frequency of vector-host fusion transcripts, so we performed a basic analysis of cellular fusion transcripts.

HEK293T cells were transduced with CCL-GEW, LTR1.7.671-GEW, or LTR1.20-GEW at a range of doses and expanded for 14 days to deplete any unintegrated proviruses. VCNs were calculated by qPCR (3.43 for CCL, 2.82 for LTR1.7.671, and 3.62 for LTR1.20). Total RNA was extracted from transduced cells and ribosomal RNA was depleted prior to preparation of next-generation sequencing libraries. Sequencing reads were analyzed for fusion transcripts by mapping paired reads to the human genome and subsequently to the relevant vector provirus, to find the frequency of human-vector transcript chimera per human genomic transcript. The number of fusion transcripts per 10^6 human transcripts was then normalized to the dsDNA provirus copy number in each sample.

The fusion transcript frequency detected in CCL samples (0.54 ± 0.06) was significantly greater than LTR1.7.671 samples (0.07 ± 0.02) and LTR1.20 samples (0.14 ± 0.02) (p = 0.027 by Kruskal-Wallis test) (Figure 4B). These values were also expressed relative to the number of CCL fusion transcripts, to highlight the reduced frequency of splice fusions when using LTR1.7.671 (12.97 ± 5.64% of CCL level)

and LTR1.20 (26.5 ± 0.86% of CCL level) ($p = 0.024$ by Kruskal-Wallis test) (Figure 4C).

LTR1 Vectors Can Exceed Standard Lentiviral Vector Efficacy in the Liver following Murine Neonatal Intravenous Injection of Titer-Matched Viruses

To confirm that expression from an LTR1 provirus was detectable in vivo, LTR1.20-SFFV-EGFP (titer 2.34×10^7 TU/mL) was produced and administered intracranially or intravenously to newborn CD1 mice (Figure 5). Examination of EGFP expression 1 week after vector administration demonstrated that LTR1 could efficiently deliver transgene expression to mouse liver and brain (Figure 5A). Immunostaining of dissected brains demonstrated that stable EGFP expression existed predominantly within the cortex and hippocampus of the injected hemisphere (Figure 5B).

To compare longitudinal LTR1-driven in vivo expression to a CCL vector, the SFFV-Luc-T2A-EGFP bicistronic construct was used to allow bioluminescence imaging. Equivalent volumes of lentiviral vectors (LTR1.20 titer of 2.0×10^7 TU/mL and CCL titer of 3.6×10^7 TU/mL) were administered either intracranially or intravenously to newborn CD1 mice, which were then monitored for 36 days.

In vivo LTR1.20-SFFV-Luc-T2A-EGFP bioluminescence imaging revealed that the brain expression profiles of LTR1.20 did not show a statistically significant difference to CCL vectors ($p = 0.087$) (Figure 5C). However, comparison of luciferase expression in the livers of intravenously injected animals showed a significantly higher bioluminescent output from LTR1 vectors over the 36 day period ($p = 0.018$) (Figure 5D). The biodistribution and intensity of luciferase expression is shown in representative images at 0, 5, 15, and 36 days post-administration in Figure S7.

The Onset of Transgene Expression from LTR1 Vectors Occurs Earlier than Third Generation Lentiviral Vectors

An interesting observation was made when imaging the SFFV-Luc-T2A-EGFP animals immediately after vector administration. At just 20 min post-injection, luciferase expression was detectable in all LTR1.20-treated animals, but not in animals injected with third generation CCL vectors (Figure 6A).

In light of this finding, we sought to profile the timing of LTR1-derived expression during the initial stages after transduction. HT1080 cells were transduced at a MOI of 1 with LTR1.11.1-GEW, LTR1.13.0-GEW, or CCL-GEW (vector schematics can be found in Figure 2A). Flow cytometric analysis during the initial 48 hr after transduction revealed that the percentage of EGFP-positive cells derived from the LTR1.13.0 backbone was already at 8% just 1 hr after transduction, increasing at each subsequent time point (Figure 6B). Expression from the LTR1.11.1 and CCL backbones, which each contain a 5′LTR in their leader sequence, was minimal until 8 hr after transduction and increased thereafter. The percentage of EGFP-positive cells produced by LTR1.13.0 was significantly higher than LTR1.11.1 and CCL over the duration of the experiment, as determined by the differences between AUCs ($p = 0.039$ by Kruskal-Wallis multiple comparison test).

We looked deeper into the rapid onset of LTR1 expression by examining the timing of intracellular reverse-transcription products using late-RT qPCR[35] (Figure 6C). This analysis revealed that LTR1.13.0 reverse-transcription products were significantly greater than CCL over the initial 6 hr post-transduction ($p = 0.036$ by t test). LTR1.13.0 late-RT products were detectable by 1 hr post-injection, at a level not matched by CCL until 4 hr post-injection (Figure 6C). LTR1.11.1 did not produce late-RT products significantly faster than CCL ($p = 0.057$), suggesting that minus-strand transfer may be an influential factor in the onset of LTR1 expression.

LTR1 Vectors Can Be Used to Correct a Factor IX-Deficient Mouse Model

To demonstrate gene therapy with LTR1 technology, we sought to correct a factor IX (FIX)-deficient mouse model of hemophilia B by in vivo FIX gene transfer. For this, we used codon-optimized factor IX cDNA (FIX) containing the hyperactive Padua mutation.[36] To provide sufficient vector yields for this experiment, LTR1.25 and LTR1.27 backbones were used alongside the conventional CCL vector backbone for comparison of in vivo transduction. CCL-SFFV-FIX, LTR1.25-SFFV-FIX, and LTR1.27-SFFV-FIX vectors were titered by qPCR to determine the infectious dsDNA proviral copy number.

Vectors were delivered to neonatal mice by intravenous administration at post-natal day 1. The vector doses were determined by the total number of administered viral genomes, with doses being 1.4×10^8 dsDNAvg for CCL, 1.7×10^7 dsDNAvg for LTR1.25, and 1.5×10^8 dsDNAvg for LTR1.27. Mouse livers and plasma samples were collected upon termination of the experiment. The liver proviral copy number was determined by qPCR, which were calculated as being 1.8 ± 0.9 for LTR1.25, 1.7 ± 0.9 for LTR1.27, and 1.4 ± 0.5 for CCL (Figure 7A).

LTR1.25-treated mouse plasma contained mean factor IX protein levels of 12.2 ± 5.2% of normal reference levels, which was similar to the 12.3 ± 3.7% produced by CCL (Figure 7B). Mean plasma factor IX activity was raised to 14.9 ± 7.5% normal levels by LTR1.25, which again matched the CCL mean output of 12.6 ± 4.0% (Figure 7C). LTR1.27 treatment gave 22.9 ± 1.7% factor IX protein levels and 24.4 ± 3.0% factor IX activity. No statistically significant difference was detected during analysis of plasma factor IX protein ($p = 0.153$ by Kruskal-Wallis) and activity ($p = 0.35$ by Kruskal-Wallis). The overall level of factor IX restoration would be sufficient for corrective gene therapy in humans,[37,38] thus demonstrating that LTR1 is effective as a gene therapy vector in this setting.

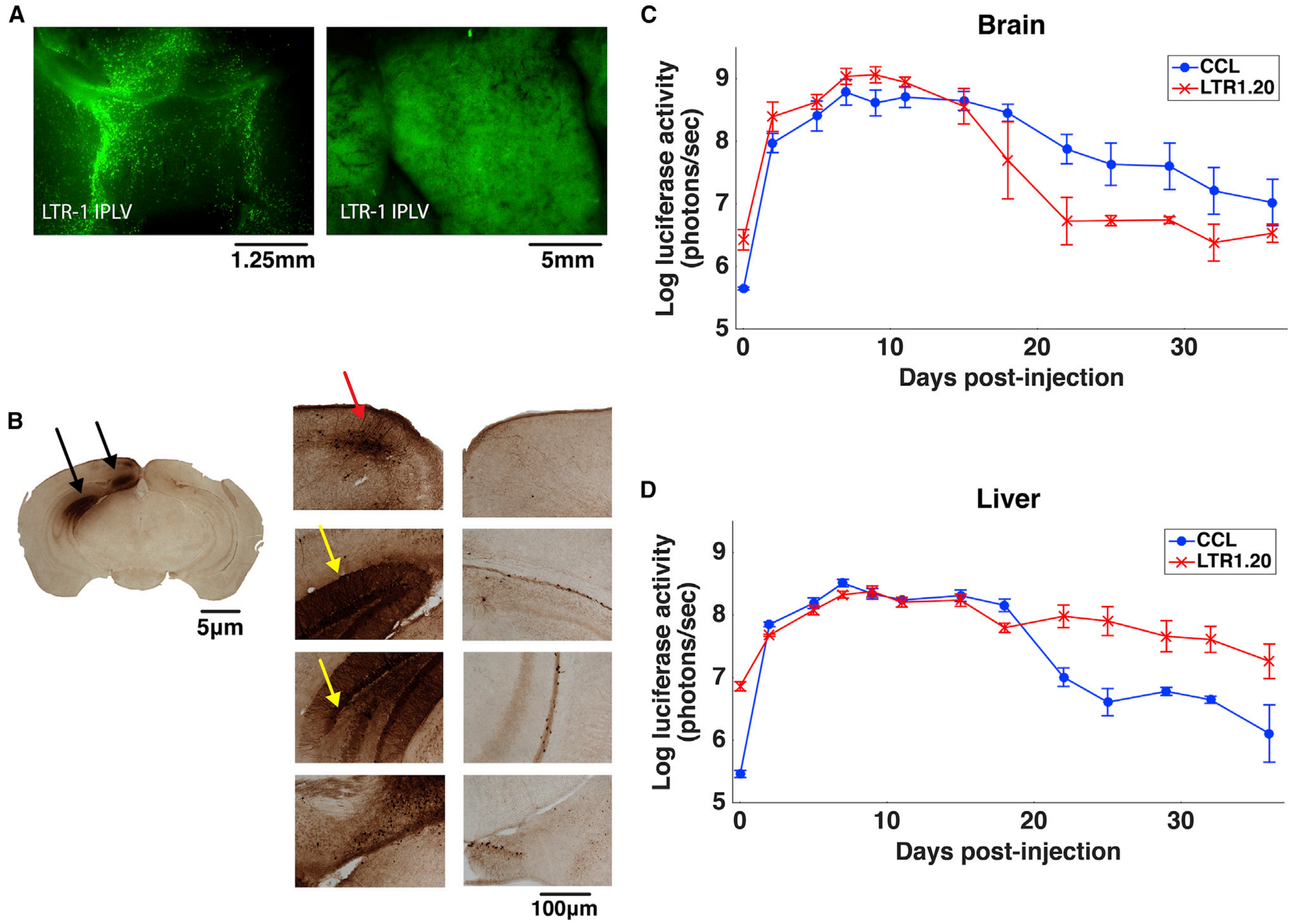

Figure 5. LTR1 Vectors Can Be Used for Efficient In Vivo Gene Delivery

(A) Newborn mice were either intracranially or intravenously injected with integration-proficient (IPLV) LTR1.20-SFFV-EGFP to target brain and liver, respectively. After 1 week, macro fluorescence microscopy revealed strong EGFP expression in both organs. (B) Immunohistochemical staining of paraffin-embedded coronal slices of injected brains shows neuronal EGFP expression following intracranial LTR1 delivery. The black arrows show areas with EGFP positivity, while the yellow and red arrows show hippocampal and cortex staining, respectively. (C) Longitudinal bioluminescent monitoring of LTR1 and CCL luciferase expression in the brain. Newborn mice were intracranially injected at post-natal day 1 with either CCL-SFFV-Luc-T2A-EGFP or LTR1.20-SFFV-Luc-T2A-EGFP (n = 3 animals for each group, data expressed as means ± SEM). Brain bioluminescence was quantified at regular time intervals over a 36 day period. No statistically significant difference was detected by t test ($p = 0.087$), indicating that LTR1.20 is capable of matching standard lentiviral vector brain transduction. (D) Bioluminescent monitoring of LTR1 expression in the liver following intravenous vector injection at post-natal day 1 with either CCL-SFFV-Luc-T2A-EGFP or LTR1.20-SFFV-Luc-T2A-EGFP (n = 3 animals for each group, data expressed as means ± SEM). Bioluminescent measured in LTR1.20-transduced livers was significantly higher than CCL-transduced livers over the entire 36 day period following t test comparison of area under the curves ($p = 0.018$).

DISCUSSION

Lentiviruses offer a number of advantages as gene therapy vectors and their development continues to progress.[3–6] They can be engineered to carry genetic information into a broad range of cell types and can efficiently integrate their genomes into target cell chromosomes, resulting in permanent DNA delivery. These properties make lentiviral vectors useful in a range of scenarios, such as studying the biological effects of transgenes on pre-clinical disease models, the generation of transgenic animal strains, and the transfer of therapeutic sequences to treat human disease.[39,40] Despite these advantages, there is scope for further improvement of lentiviral vector technology, given their reported interference with the human genome owing to the concurrent delivery of wild-type HIV *cis* elements.[19,20,41,42]

We have developed a lentiviral vector with a novel formation that ensures minimal transfer of wild-type HIV-1 sequences. The LTR1 reverse-transcription mechanism is unprecedented in retrovirology, as all other retroviruses and retrovirus-derived vectors require at least two strand transfer events to complete proviral synthesis. In LTR1 vectors, minus-strand synthesis is primed adjacent to a solitary 3′LTR, thus rendering the usual first strand transfer event obsolete. This demonstrates that the first strand transfer event in reverse-transcription is not necessary for functional lentiviral transduction of

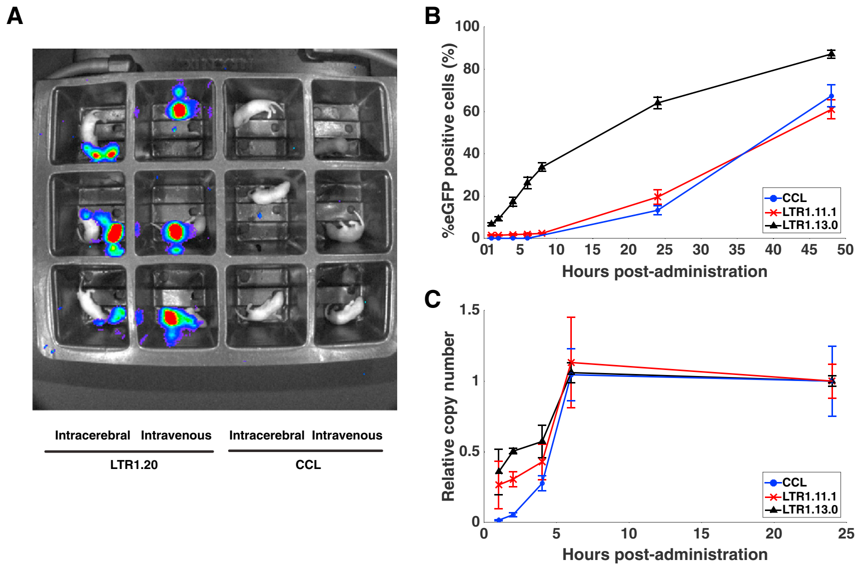

Figure 6. LTR1 Vectors Produce Transgene Expression Earlier than Standard Lentiviruses

(A) Newborn mice were imaged 20 min after intracranial or intravenous injection of CCL-SFFV-T2A-EGFP or LTR1.20-SFFV-T2A-EGFP to determine whether any early expression was detectable. LTR1 vectors are capable of producing very early transgene expression, which is not seen with third generation CCL. (B) Time course experiment showing the timing of EGFP expression during the initial 48 hr after transduction. HT1080 cells were plated and simultaneously transduced with CCL-GEW (blue line, circle marker), LTR1.11.1-GEW (red line, cross marker), or LTR1.13.0-GEW (black line, triangle marker) at MOI 1 (n = 3 for each group, data expressed as means ± SEM). Transduced cells were harvested at various time points and processed in a flow cytometer to determine the percentage of EGFP-positive cells. The LTR1.13.0 vector, which does not contain a 5′LTR, began to produce EGFP expression at an earlier time point and sustained higher expression than CCL or LTR1.11.1 vectors, which do contain a 5′LTR ($p = 0.039$ by Kruskal-Wallis multiple comparison of area under the curves for the three experimental groups). (C) Analysis of reverse-transcription products in HT1080 cells during the initial 24 hr post-transduction shows that LTR1.13.0 and LTR1.11.1 products appear sooner than those of CCL. Reverse-transcription products are expressed relative to the final copy numbers detected at 24 hr post-transduction (n = 3 for each group, data expressed as means ± SEM).

target cells, revealing an interesting facet of HIV biology. Importantly, this potentially constitutes a shortening of the viral life-cycle and increased in vivo efficacy, analogous to self-complementary AAV (scAAV).[43]

The structure of the LTR1 provirus reveals interesting insights into HIV biology and brings an important technological advancement in terms of the potential safety of clinical gene therapy. HIV-1 RNA packaging sequences comprise approximately 1.9 kb of its genome, meaning that third generation lentiviruses bear 20% sequence homology with wild-type HIV-1. Here, and in previous studies, lentiviral proviruses have been shown to produce full-length RNA genomes that can be remobilized in replicating HIV-1 particles, even with the use of a self-inactivating LTR.[22–24] Third generation vector genome remobilization poses a risk that cannot be ignored when using standard third generation vectors, but, importantly, we show that it can be avoided through use of LTR1. In our investigations, HIV-1 packaging sequences were undetectable in LTR1 proviruses, which were resistant to remobilization in the HIV-1-like particles, thus providing a significant advancement in clinical safety. This is important when considering lentiviral vectors as agents for treatment of HIV-1 infections and, as the number of trials using lentiviruses increases and becomes more widespread, this will concurrently increase the risk of co-infection with HIV.

HIV-1 packaging sequences contain active splice donor and splice acceptor sites, which have previously proven very difficult to remove from vector genomes.[27–29] We performed a preliminary investigation into the frequency of vector-host fusion transcripts in HEK293T cells transduced with LTR1 and standard lentiviral vectors. Data resulting from these initial analyses showed that LTR1 technology can reduce the frequency of splice fusions by 87%, compared to standard lentiviral vectors. LTR1.20 fusion transcripts were slightly higher than LTR1.7.671, possibly due to the presence of an intron in the LTR1.20 leader sequence, but still showed a 73.5% reduction from the CCL level. The reduced propensity of LTR1 to splice into cell genes would enhance clinical safety by reducing the risk of generating potentially harmful patient-vector fusion transcripts. This would bring significant advantages for the long-term clinical use of lentiviral vectors, given the reported links between retrovirus-host fusion transcripts and oncogenesis and warrants further investigation in the future.[20,41,44,45]

We have shown that LTR1 vectors can be produced at titers sufficient for pre-clinical gene therapy. We demonstrated LTR1-mediated correction in a hemophilia B mouse model, in which LTR1.25 and LTR1.27 were able to deliver liver VCNs equal to a standard CCL lentiviral vector. Plasma factor IX expression was detected at therapeutic levels and with sufficient biological activity, upon termination of the experiment, indicating that LTR1 could be used for stable disease correction in a gene therapy setting.

Close analysis showed that LTR1.27 restored mouse plasma factor IX activity to around 24% of normal levels when delivered at a similar dose to CCL, which returned around 13%. The level of factor IX expression derived per injected LTR1.27 genome is an intriguing matter for further investigation. While encouraging, it cannot be ignored that all vector doses were based on the infectious titer, which we have identified as being lower than the physical particle titer in the

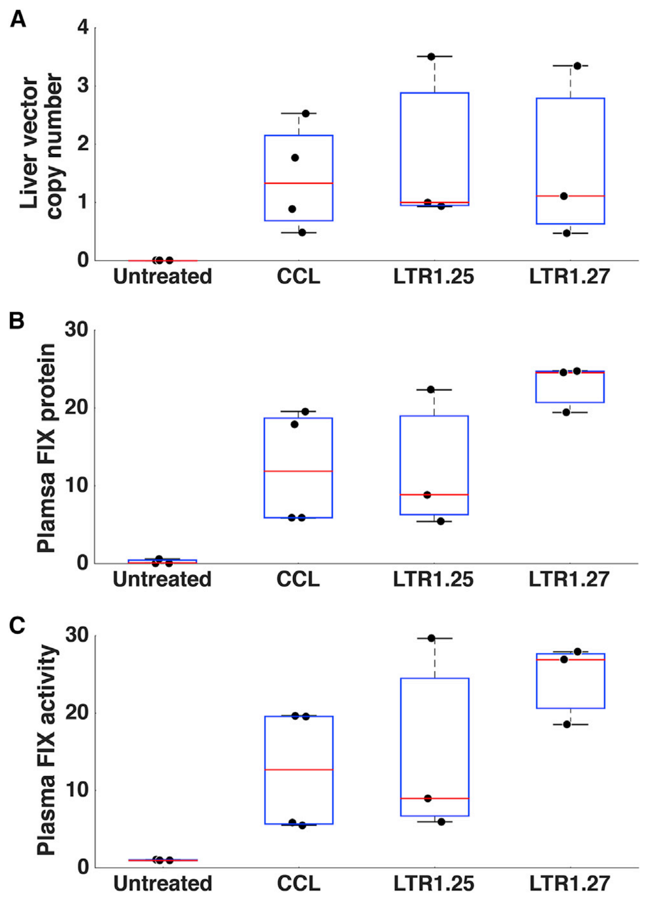

Figure 7. LTR1 Increases Plasma Factor IX Expression following Treatment of a FIX-Deficient Mouse Model

LTR1.25-SFFV-FIX (n = 3), LTR1.27-SFFV-FIX (n = 3), or CCL-SFFV-FIX (n = 4) was intravenously administered to newborn mice and compared to untreated knockouts (n = 3). Plasma factor IX was assayed when the experiment was terminated. All data are presented as individual data points with overlaid boxplots representing median lines (red), the upper and lower range (black lines), and 75% confidence intervals (blue boxes). (A) qPCR calculation of liver proviral copy numbers found that LTR1.25, LTR1.27, and CCL liver transduction was comparable. (B and C) Plasma factor IX protein levels (% normal levels) (B) and plasma factor IX activity levels (% normal levels) were restored to therapeutic levels by all vector treatment groups (C).

case of LTR1.20, compared to CCL. Therefore, there is a possibility that excess defective particles were also injected along with functional LTR1.25 and LTR1.27 particles, potentially impacting on transduction. Possible mechanisms might include blocking clearance of particles in cells such as Kupffer cells, thus allowing more vector particles to infiltrate hepatocytes.

During longitudinal tracking of luciferase expression in neonatally injected CD1 mice, LTR1 showed significantly more liver bioluminescence than a titer-matched third generation CCL over the course of the experiment (36 days). The exact mechanism for this difference is unclear, and the influence of excess defective particles cannot be ruled out, considering that the CCL profile appears to match LTR1 at early stages of the investigation before decreasing from day 18, which was less pronounced with LTR1. This profile was not observed following brain transduction.

An intriguing feature witnessed during LTR1 characterization was the rapid onset of transgene expression. This was observed both in vitro and in vivo, with two separate vector transgene cassettes. Following intracerebral and intravenous LTR1.20-luciferase injections, mice were immediately examined for bioluminescence. This investigation revealed that LTR1.20 was capable of producing luciferase expression 20 min after injection, potentially indicating a unique functionality that could be exploited. This early level of expression was not provided by third generation CCL. Leading on from this finding, we sought to investigate how the structure of LTR1 RNA could influence the timing of transgene expression in vitro. This experiment revealed that when LTR1 was lacking a 5′LTR (LTR1.13.0; Figure 2A), there was a significant acceleration of transgene expression during the initial 48 hr after transduction. Again, this may relate to the scAAV vector paradigm in which shortening of the replication phase of transduction improves the speed and efficiency of transgene expression.[43] Given that the percentage of EGFP-positive cells increased across every time point, we would expect that any delivery of pseudo-packaged EGFP protein[46] or direct translation of vector RNA[47] would have had a negligible effect on total EGFP output.

The data concerning the onset of reverse-transcription products suggest that the smaller LTR1 genome and unique reverse-transcription pathway may facilitate rapid copying of its RNA genome, thus accelerating the onset of gene expression. Interestingly, LTR1.11.1, which does perform both strand transfer events, also produced reverse-transcription products earlier than a standard vector, suggesting that minus-strand transfer is not the exclusive reason for differences in the speed of transduction. The size of the LTR1.11.1 provirus is approximately 1.5 kb smaller than that of the standard CCL vector, thus the speed of transduction may be partly influenced by the size and complexity of the template to be copied. This rapid onset of expression constitutes a unique feature of LTR1 that may be exploited for gene therapy purposes in scenarios requiring vector expression within a short time frame. This could be particularly advantageous during ex vivo manipulation of stem cells and when using non-integrating lentiviral vectors, given that unintegrated proviruses are rapidly lost from dividing cells after repeated passages.

Our extensive characterization of LTR1 vectors has revealed interesting features that will be important to consider when moving forward with further LTR1 development. During northern blot analysis of packaged vector genomes, we discovered that LTR1 vectors are dependent on the expression of HIV-1 Tat in producer cells for efficient transcription of full-length viral genomes. We observed that shorter transcripts were generated in the absence of Tat, presumably due to undesirable termination of transcription and poly-adenylation at the solitary LTR. We expect that this was rescued by Tat due to its ability to promote transcriptional readthrough of the 3′LTR through its binding the HIV-1 trans-activation response element (TAR).[48]

Additionally, we have highlighted that in vitro transduction of cells requires a greater LTR1.20 p24 dose and vector RNA dose to match the efficacy of a standard lentiviral vector. LTR1.20 RNA titers and physical particle titers were calculated as being similar to CCL, suggesting that RNA packaging is not compromised. However, the reduced titer of integrated proviruses and GFP-forming units suggest that differences may exist in the processing of LTR1.20 RNA during transduction and integration. The later generations of LTR1.25 and LTR1.27 show improved titers, hinting at an improved level of stable transduction efficiency. As LTR1 technology continues to progress, it is possible that the efficiency of transduction will eventually match that of standard lentiviral technology. By understanding and controlling LTR1 characteristics, there is potential to further increase the infectious titer through experimentation and process development to exploit its unique characteristics. Furthermore, if integration efficiency does prove to be the limiting factor for in vitro LTR1 efficacy, then LTR1 could be expected to meet the efficiency of standard lentiviral technology when used as an integration-deficient lentivirus (IDLV). Until the precise mechanism for limited LTR1 infectivity is defined, we must acknowledge the risk that the presence of defective LTR1 particles might create, which could theoretically cause adverse effects in humans. Furthermore, a low infectious titer versus a physical titer could negatively impact on the feasibility of licensed LTR1 products.

Gene therapy with lentiviral vectors is being pursued for a rapidly increasing cadre of diseases including beta globinopathies, chronic granulomatous disease, leukodystrophies, and blood cancers.[49–54] As the range of targets broadens and larger patient populations stand to benefit, it will be necessary to ensure that vectors provide the maximum assurance of patient safety. LTR1 vector technology offers a safety improvement that meets this need and could form an important component of next-generation lentiviral vectors.

MATERIALS AND METHODS

Generation of Plasmid Constructs

All plasmid constructs were made using standard molecular cloning procedures and PCR-mediated deletion of plasmid sequences.[55] Detailed plasmid information is available upon request.

Cell Culture Maintenance

HEK293T cells were used for production of viral vectors, remobilization assays, and for analysis of fusion transcripts. HT1080, a human fibrosarcoma cell line, was used for titration of VCNs by qPCR. HEK293T and HT1080 cell lines were cultured at 37°C in DMEM (Invitrogen) supplemented with 10% (v/v) fetal bovine serum. All lines were split three times per week, when ~80% confluence was reached.

Production of Lentiviral Vectors

Lentiviruses were produced using a second-generation packaging system as described previously.[3] Briefly, 1.8×10^7 HEK293T cells were plated in a 15 cm dish and transfected with 40 μg of the relevant transfer plasmid, 30 μg of pCMVΔR8.74, and 10 μg of pMDG.2 DNA (plasmids produced by PlasmidFactory) and were mixed in 5 mL Opti-MEM and filtered through a 0.22 μm filter before combining with 5 mL Opti-MEM (Life Tech/GE) containing 1 μL of 10 mM polyethylenamine (PEI, Sigma). The resulting 10 mL mixture was applied to HEK293T cells after 20 min incubation at room temperature. The transfection mix was removed after 4 hr and replaced with fresh culture medium. Virus-containing medium was collected twice at 48 hr and 72 hr post-transfection. After each harvest, the collected medium was filtered through a cellulose acetate membrane (0.45 μm pore). Lentivirus harvests were combined before concentration by ultracentrifugation. Briefly, viruses were placed in 25 × 83 mm polyallomer centrifuge tubes (Beckman Coulter) and centrifuged for 2 hr at 90,000 × *g* at 4°C in a Sorvall Discovery 90SE Centrifuge. Following centrifugation, the supernatant was removed and the pellet was re-suspended in 200 μL Opti-MEM for 500-fold concentration.

Titration of Lentiviral Vectors

Vector titration by flow cytometry: 1×10^5 HEK293T cells were plated into each well of a 6-well plate and transduced with a range of volumes of concentrated lentivirus. At 72 hr after transduction, cells were trypsinized and EGFP-positive cells were quantified using a BD Cyan flow cytometer or BD FACSArray Bioanalyzer 3 days after transduction.

HEK293T cells were not used for qPCR titration due to their reported abnormal karyotype.[56] Briefly, 1×10^5 HT1080 cells were plated into each well of a 6-well plate and transduced with a range of volumes of concentrated lentivirus. At 72 hr after transduction, genomic DNA was extracted and the provirus titer calculated by qPCR, as described previously.[57] The viral capsid number was determined using a p24 ELISA kit (Clontech - 632200). The capsid number was determined according to the kit manufacturer's calculations, where 1 ng p24 is equivalent to 1.25×10^7 lentiviral particles (lp). The vector RNA genome titer was determined using a qRT-PCR RNA titration kit containing pre-designed primers and standards (Clontech - 631235). For all titer comparisons, LTR1 and third generation vectors were produced side-by-side to account for variations between production batches.

Detection of EGFP Expression in Transduced Cells

Flow cytometric detection of EGFP expression was used for titration and characterization of LTR1 vectors. Unless stated otherwise, 100,000 cells were analyzed for detection EGFP expression in a BD FACSArray Bioanalyzer. EGFP fluorescence was excited using a 488 nm laser. During analysis of flow cytometry plots, cells were gated by plotting forward-light-scatter versus side-scatter to isolate the live population. EGFP-positive populations were identified by plotting EGFP fluorescence (detected using a 530/30 nm band-pass filter) versus emission from the yellow channel (detected using a 575/26 band-pass filter) to compensate for auto-fluorescent events. Non-transduced cell populations were used as negative controls to set the background level of emission in each channel. All flow

cytometry data were analyzed by FlowJo software version 9.3.1 (Tree Star).

Dose-Response Profiling of LTR1 Vectors versus Third Generation Lentiviral Vectors In Vitro

HT1080 cells were infected with either LTR1.20-SFFV-Luc-T2A-EGFP or CCL-SFFV-Luc-T2A-EGFP at a range of MOI, prepared by serial 2-fold dilutions of starting stocks. To avoid the influence of unintegrated lentiviral expression, cells were expanded for 14 days before analysis by flow cytometry and genomic DNA extraction for proviral copy number analysis. The starting vector stock was assayed for p24 concentration by p24 ELISA and the vector RNA genome titer was calculated by qRT-PCR, with methods described in Titration of Lentiviral Vectors.

PCR Analysis of Integrated LTR1 Proviruses

HT1080 cells were transduced with either LTR1.20-SFFV-EGFP or CCL-SFFV-EGFP at an MOI of 10. At 2 weeks after transduction, cells were harvested and genomic DNA was extracted using a DNeasy Blood and Tissue kit (QIAGEN). To determine the size of the vector backbone, genomic DNA was PCR-amplified using oligos specific for the lentiviral 5′LTR-primer binding site junction (5′-AAATCTCTA GCAGTGGCGCCCGAACAG-3′) and the 3′LTR R region (5′-GCA CTCAAGGCAAGCTTTATTGAGGCTT-3′).[58] The PCR was carried out using q5 polymerase (New England Biolabs) with conditions: 98°C for 30 s; followed by 28 cycles of 98°C for 10 s and 72°C for 1 min 50 s; and a final step of 72°C for 2 min. Amplicons were visualized on a 1% agarose gel to confirm provirus sizes.

Northern Blotting of Vector RNA

RNA species from pCCL, pLTR.1.7.671, and pLTR1.20 were analyzed during particle production or after proviral integration. For analyzing vector genomes during packaging, 10^7 HEK293T cells were transfected with 12 μg pcDNA3.HIV-1g/p.4×CTE,[59] 5 μg pRSV.Rev (kindly provided by T.J. Hope, Chicago, IL), 5 μg pcDNA3.Tat, 2 μg pMD2.G, and 5 μg pCCL, pLTR.1.7.671, or pLTR1.20 vector DNA using the calcium phosphate transfection method. Transfection was controlled by co-transfecting 2 μg non-viral pCMV.DsRedexp expression plasmid (Clontech Takara). At 2 days post-transfection, the cells were harvested, analyzed by flow cytometry and their total RNA was extracted using RNAzol-RT (Molecular Research Center) according the manufacturer's instruction. For the analysis of proviral vector RNA species, 2×10^5 HEK293T cells were transduced with serial dilutions of CCL, LTR.1.7.671, or LTR1.20. At 4 days post-transduction, cells were harvested, analyzed by flow cytometry, and the total RNA of similarly transduced cells (59%–65% $EGFP^+$) was extracted with RNAzol-RT. All isolated RNAs were subjected to northern blot analysis using standard protocol procedures. In brief, 10 μg of each RNA sample were separated on 1% agarose gel under denaturing conditions, transferred to a Biodyne B membrane (Pall) and analyzed via radioactive probing. Probes directed against EGFP and 18S RNA (loading control) were labeled with 32P using the DecaLabel DNA Labeling Kit (Thermo Fisher Scientific).

Examination of Provirus Structure by Plasmid Rescue

The CCL-Rescue and LTR1.20-Rescue plasmids were produced by excising the pBR322[60] elements from the respective plasmids and relocating them to the lentiviral transgene region (Figure S4). HEK293T cells were plated in 6-well plates at a density of 1×10^5 cells per well and transduced with either 10 μL (0.1 μg p24) of concentrated CCL-Rescue or 200 μL (15 μg p24) of concentrated LTR1.20-Rescue. Cells were maintained in culture for 2 weeks before extracting genomic DNA. For each sample, 10 μg of genomic DNA was treated with XbaI (to ensure extensive cutting of the human genome while avoiding digestion of viral genomes) for 1 hr before column purification (QIAGEN PCR Purification Kit - 28104) and ligation. Electrocompetent Stbl4 cells (Life Technologies) were transformed with the ligated sample in a 0.1 mm electroporation cuvette at a frequency of 1.2 kHz and 25 μF capacitance. Transformed bacteria were then selected on agar plates (100 μg/mL ampicillin) to isolate any provirus-containing colonies, from which plasmid DNA was harvested and screened for the presence of lentiviral proviruses by targeted restriction digest of lentiviral LTRs (with AflII) extracted plasmid DNA. The provirus-containing plasmids were subsequently sequenced to determine the composition of integrated LTR1.20 and CCL proviruses.

Detection of Remobilized Self-Inactivating Lentiviral Genomes

HEK293T cells were transduced with CCL-SFFV-EGFP or LTR1.20-SFFV-EGFP with a range of vector doses. At 11 days after transduction, a sample of each population was taken for genomic DNA extraction and qPCR quantification of VCNs. For each production replicate, 1.8×10^7 transduced cells were seeded into T175 flasks. At 24 hr later, each flask was transfected with 30 μg of pCMVΔR8.74 and 10 μg of pMDG.2. DNA was mixed in 5 mL Opti-MEM and filtered through a 0.22 μm filter before combining with 5 mL Opti-MEM containing 1 μL PEI (10 mM). The resulting 10 mL mixture was applied to the transduced cells after 20 min incubation at room temperature. The transfection mix was removed after 4 hr and replaced with fresh culture medium. Lentiviral supernatants were processed as described in Production of Lentiviral Vectors. Fresh HEK293T cells were seeded in 6-well plates at a density of 1×10^5 cells per well and transduced with 50 μL of the concentrated viruses (n = 3). At 11 days after transduction, cells were analyzed by flow cytometry to detect the number of EGFP-expressing cells. Cells were analyzed in a BD FACSArray Bioanalyzer and non-transduced cells provided the baseline for background fluorescence.

Transcriptomic Profiling of Vector-Host Fusion Transcripts

HEK293T cells were transduced with CCL-GAPDH-EGFP, LTR1.7.671-GAPDH-EGFP, or LTR1.20-GAPDH-EGFP at a range of doses and VCNs quantified by qPCR. Samples with similarly matched copy numbers were processed for transcriptomic profiling. Total RNA was extracted from cells and 1 μg was processed, with ribosomal RNA depleted using the Kapa RiboErase kit (Kapa Biosystems - KK8483). RNA was fragmented to produce intact fragments of 200–300 bp and adaptor-ligated sequencing libraries were prepared according to the manufacturer's protocol.

Libraries were sequenced on the Illumina NextSeq system. FASTQ files were analyzed on the Galaxy platform[61] (http://www.usegalaxy.org instance) by mapping paired reads to the human genome (hg38) using HISAT[62] and subsequently BWA-MEM[63] mapping the pair of hg38-aligned reads to the relevant vector provirus.

Animal Procedures

For in vivo investigations, outbred CD1 mice (Charles River), or hemophilia B mice[64] were time mated to produce neonatal animals. At post-natal day 1, non-randomized neonates were subjected to brief hypothermic anesthesia and injected with lentiviral vectors via the appropriate route. Intracranial injections were performed bilaterally into the lateral ventricles.[65] Intravenous injections were administered via the superficial temporal vein. Experimental groups were blinded during the course of in vivo investigations, with the exception of images taken for Figure 6A. All experiments were performed in accordance with relevant guidelines and regulations. Experiments were carried out under United Kingdom Home Office regulations and approved by the ethical review committee of University College London.

Confirmation of Vector Efficacy In Vivo by Luciferase Expression

To monitor LTR1 bioluminescence in vivo, LTR1.20-SFFV-Luc-T2A-EGFP (2.0×10^7 TU/mL) or CCL-SFFV-Luc-T2A-EGFP vectors (3.6×10^7 TU/mL) were administered either intracranially (2×5 μL bilaterally) or intravenously (40 μL) to 1-day-old neonatal CD-1 mice. Images and bioluminescence data were gathered continually for 36 days as described previously.[34] Briefly, animals were intraperitoneally injected with firefly D-luciferin (150 mg/kg) and imaged after 5 min with a cooled charge-coupled device (CCD) camera (IVIS Lumina II, PerkinElmer). Regions of interest were defined manually using a standard area for the organ under investigation. Signal intensities were expressed as photons per second per centimeter^2 per steradian, with background signal measured at each time point and subtracted from test values.

Immunohistochemistry Staining and EGFP Imaging of LTR1.20-SFFV-EGFP Transduced Brains

CD-1 mice were injected with LTR1.20-SFFV-GFP (2.34×10^7 TU/mL) at post-natal day 1. Mice received vector either by direct intracranial injection into the left lateral ventricle (5 μL), or intravenously (20 μL). At 1 week later, they were sacrificed and organs imaged by fluorescence microscopy (Leica MZ16) or immunohistochemistry. For immunohistochemistry, brains were embedded in paraffin wax and sliced in the coronal plane in preparation for EGFP-staining. Brain sections were treated with 30% H_2O_2 in tris-buffered saline (TBS) for 30 min and blocked with 15% of goat serum (Vector Laboratories) in TBS-tween 20 (TBST) for 30 min. Rabbit anti-GFP (1:10,000; Abcam) was added in 10% goat serum in TBST and left on a gentle shaker overnight at 4°C. Goat anti-rabbit (1:1,000; Vector Laboratories) was then added in 10% goat serum in TBST for 2 hr. The sections were incubated for a further 2 hr with VECTASTAIN ABC (Vector Laboratories), followed by addition of 0.05% 3,3′-diaminobenzidine (DAB) and brief incubation. Sections were transferred to ice-cold TBS. Each individual brain section was mounted on chrome gelatin-coated Superfrost Plus slides (VWR) and left to dry for 24 hr. The slides were dehydrated in 100% ethanol and placed in Histo-Clear (National Diagnostics) for 5 min before mounting with DPX mounting medium (VWR).

Monitoring In Vitro EGFP Expression during Early Stages of LTR1 Transduction

HT1080 cells were plated onto 6-well plates at a density of 1×10^5 cells per well in a 1 mL volume and simultaneously transduced with LTR1.11.1-GAPDH-EGFP, LTR1.13.0-GAPDH-EGFP, or CCL-GAPDH-EGFP at an MOI of 1. At various time points after transduction, cells were trypsinzed and harvested for flow cytometric analysis of EGFP expression as described in Titration of Lentiviral Vectors. Genomic DNA was extracted from cell pellets and analyzed for reverse-transcription products using a previously reported late-RT qPCR assay.[35] Briefly, genomic DNA was treated with DpnI restriction enzyme to remove any plasmid carried over during vector production, before qPCR analysis by absolute quantification. Copy numbers were extrapolated from a standard curve and copy numbers were expressed relative to the copy number detected at 24 hr post-transduction.

LTR1-Mediated FIX Delivery

A codon-optimized version of FIX containing the hyperactive Padua mutation[36] was synthesized by Integrated DNA Technologies and cloned into pLTR1.25-SFFV, pLTR1.27-SFFV-FIX, or pCCL-SFFV using AgeI and SalI restriction sites. Either CCL-SFFV-FIX (dose of 1.4×10^7 dsDNAvg/mL) ($n = 4$), LTR1.25-SFFV-FIX (dose of 1.7×10^7 dsDNAvg/mL) ($n = 4$), or LTR1.27-SFFV-FIX (dose of 1.7×10^7 dsDNAvg/mL) ($n = 3$) was intravenously administered to factor IX-deficient mice at post-natal day 1 ($n = 4$ each). Blood samples were collected by lateral tail-vein puncture upon termination of the experiment (67 days for CCL and LTR1.25 and 37 days for LTR1.27). Plasma samples were analyzed for FIX expression by ELISA (VisuLize Factor IX Antigen Kit - Affinity Biologicals) and for FIX activity by chromogenic assay (BIOPHEN Factor IX chromogenic assay - Aniara). During the investigation, one untreated mouse was culled due to excessive bleeding and one LTR1.25-treated mouse was culled due to low body weight.

Statistical Analysis

All statistical analyses were carried out using either MATLAB 2015a or Python SciPy open-source software.[66] Line plots were compared by calculating the AUC by trapezoidal numerical integration and subsequently performing a statistical test on the grouped data. For multiple comparisons of EGFP percentage data, the Kruskal-Wallis test was used to compare mean AUC, as normal distribution of data was not assumed. For comparison of titrations and bioluminescence, a two-tailed Welch t test was used to compare mean AUC. Both statistical tests employed are robust for datasets without equal variance or sample size. Mouse sample sizes were limited to three or four animals per experimental group for in vivo investigations. Polynomial curve fitting for ELISA and chromogenic assays were modeled in MATLAB 2015a.

Data and Materials Availability

All LTR1 plasmids are available under a material transfer agreement (MTA) with UCL Business (mta@uclb.com).

SUPPLEMENTAL INFORMATION

Supplemental Information includes seven figures and can be found with this article online at http://dx.doi.org/10.1016/j.ymthe.2017.04.028.

AUTHOR CONTRIBUTIONS

C.A.V. and S.J.H. - design of initial concept, execution and analysis of experiments, and review of paper; J.R.C. - design, execution and analysis of experiments, and writing paper; and S.N.W., T.R.M., R.K., D.P.P., S.M.K.B., M.H.B., A.S., and M.G. - design, execution and analysis of experiments, and review of paper.

CONFLICTS OF INTEREST

C.A.V. and S.J.H. are named inventors on LTR1 technology international patent (patent application number PCT/GB2014/053102). All other authors declare no competing interests.

ACKNOWLEDGMENTS

We are grateful for the help provided by Mr. Ayad Eddaouadi at the Institute of Child Health FACS facility for his technical assistance and advice during the gathering and interpretation of flow cytometry data. We thank Dr. Tony Brooks and Dr. Mike Hubank of UCL Genomics for their assistance and advice in executing next generation sequencing experiments. We also thank Thomas Neumann at Hannover Medical School for his technical assistance in performing northern blots of vector genomic RNA. We are also grateful to Dr. Luis Apolonia of King's College London for reviewing the accuracy and reporting of HIV-1-relevant material. J.R.C. was funded by a BBSRC Follow-On Fund and MRC Confidence in Concept grant. A.S. and M.G. were supported by grants from the German Research Council (DFG/SFB738 and DFG/ REBIRTH). T.R.M. and S.N.W. were funded by NC3Rs grant NC/L001780/1. Initial work was supported by a BBSRC New Investigator Award BB/I00212X/1 (to S.J.H. and C.A.V.).

REFERENCES

1. Perkins, A.S., Kirschmeier, P.T., Gattoni-Celli, S., and Weinstein, I.B. (1983). Design of a retrovirus-derived vector for expression and transduction of exogenous genes in mammalian cells. Mol. Cell. Biol. *3*, 1123–1132.

2. Heisig, V., Jahn, G., Ebeling, M., and Laufs, R. (1989). Use of a HIV-1 retroviral vector system for gene transfer into human cells. Haematol. Blood Transfus. *32*, 406.

3. Zufferey, R., Nagy, D., Mandel, R.J., Naldini, L., and Trono, D. (1997). Multiply attenuated lentiviral vector achieves efficient gene delivery in vivo. Nat. Biotechnol. *15*, 871–875.

4. Dull, T., Zufferey, R., Kelly, M., Mandel, R.J., Nguyen, M., Trono, D., and Naldini, L. (1998). A third-generation lentivirus vector with a conditional packaging system. J. Virol. *72*, 8463–8471.

5. Zufferey, R., Dull, T., Mandel, R.J., Bukovsky, A., Quiroz, D., Naldini, L., and Trono, D. (1998). Self-inactivating lentivirus vector for safe and efficient in vivo gene delivery. J. Virol. *72*, 9873–9880.

6. Apolonia, L., Waddington, S.N., Fernandes, C., Ward, N.J., Bouma, G., Blundell, M.P., Thrasher, A.J., Collins, M.K., and Philpott, N.J. (2007). Stable gene transfer to muscle using non-integrating lentiviral vectors. Mol. Ther. *15*, 1947–1954.

7. Muesing, M.A., Smith, D.H., and Capon, D.J. (1987). Regulation of mRNA accumulation by a human immunodeficiency virus trans-activator protein. Cell *48*, 691–701.

8. Lever, A., Gottlinger, H., Haseltine, W., and Sodroski, J. (1989). Identification of a sequence required for efficient packaging of human immunodeficiency virus type 1 RNA into virions. J. Virol. *63*, 4085–4087.

9. Richardson, J.H., Child, L.A., and Lever, A.M. (1993). Packaging of human immunodeficiency virus type 1 RNA requires cis-acting sequences outside the 5′ leader region. J. Virol. *67*, 3997–4005.

10. Kim, H.J., Lee, K., and O'Rear, J.J. (1994). A short sequence upstream of the 5′ major splice site is important for encapsidation of HIV-1 genomic RNA. Virology *198*, 336–340.

11. Berkowitz, R.D., Luban, J., and Goff, S.P. (1993). Specific binding of human immunodeficiency virus type 1 gag polyprotein and nucleocapsid protein to viral RNAs detected by RNA mobility shift assays. J. Virol. *67*, 7190–7200.

12. Kutluay, S.B., Zang, T., Blanco-Melo, D., Powell, C., Jannain, D., Errando, M., and Bieniasz, P.D. (2014). Global changes in the RNA binding specificity of HIV-1 gag regulate virion genesis. Cell *159*, 1096–1109.

13. Zapp, M.L., and Green, M.R. (1989). Sequence-specific RNA binding by the HIV-1 Rev protein. Nature *342*, 714–716.

14. Daly, T.J., Cook, K.S., Gray, G.S., Maione, T.E., and Rusche, J.R. (1989). Specific binding of HIV-1 recombinant Rev protein to the Rev-responsive element in vitro. Nature *342*, 816–819.

15. Heaphy, S., Dingwall, C., Ernberg, I., Gait, M.J., Green, S.M., Karn, J., Lowe, A.D., Singh, M., and Skinner, M.A. (1990). HIV-1 regulator of virion expression (Rev) protein binds to an RNA stem-loop structure located within the Rev response element region. Cell *60*, 685–693.

16. Venkatesh, L.K., Mohammed, S., and Chinnadurai, G. (1990). Functional domains of the HIV-1 rev gene required for trans-regulation and subcellular localization. Virology *176*, 39–47.

17. Olsen, H., Nelbock, P., Cochrane, A., and Rosen, C. (1990). Secondary structure is the major determinant for interaction of HIV rev protein with RNA. Science *247*, 845–848.

18. Fischer, U., Meyer, S., Teufel, M., Heckel, C., Lührmann, R., and Rautmann, G. (1994). Evidence that HIV-1 Rev directly promotes the nuclear export of unspliced RNA. EMBO J. *13*, 4105–4112.

19. Bokhoven, M., Stephen, S.L., Knight, S., Gevers, E.F., Robinson, I.C., Takeuchi, Y., and Collins, M.K. (2009). Insertional gene activation by lentiviral and gammaretroviral vectors. J. Virol. *83*, 283–294.

20. Moiani, A., Paleari, Y., Sartori, D., Mezzadra, R., Miccio, A., Cattoglio, C., Cocchiarella, F., Lidonnici, M.R., Ferrari, G., and Mavilio, F. (2012). Lentiviral vector integration in the human genome induces alternative splicing and generates aberrant transcripts. J. Clin. Invest. *122*, 1653–1666.

21. Cavazzana-Calvo, M., Payen, E., Negre, O., Wang, G., Hehir, K., Fusil, F., Down, J., Denaro, M., Brady, T., Westerman, K., et al. (2010). Transfusion independence and HMGA2 activation after gene therapy of human β-thalassaemia. Nature *467*, 318–322.

22. Bukovsky, A.A., Song, J.P., and Naldini, L. (1999). Interaction of human immunodeficiency virus-derived vectors with wild-type virus in transduced cells. J. Virol. *73*, 7087–7092.

23. Hanawa, H., Persons, D.A., and Nienhuis, A.W. (2005). Mobilization and mechanism of transcription of integrated self-inactivating lentiviral vectors. J. Virol. *79*, 8410–8421.

24. Lucke, S., Grunwald, T., and Uberla, K. (2005). Reduced mobilization of Rev-responsive element-deficient lentiviral vectors. J. Virol. *79*, 9359–9362.

25. Podsakoff, G.M. (2001). Lentiviral vectors approach the clinic but fall back: National Institutes of Health Recombinant DNA Advisory Committee review of a first clinical protocol for use of a lentiviral vector. Mol. Ther. *4*, 282–283.

26. Manilla, P., Rebello, T., Afable, C., Lu, X., Slepushkin, V., Humeau, L.M., Schonely, K., Ni, Y., Binder, G.K., Levine, B.L., et al. (2005). Regulatory considerations for novel

gene therapy products: a review of the process leading to the first clinical lentiviral vector. Hum. Gene Ther. *16*, 17–25.

27. Cui, Y., Iwakuma, T., and Chang, L.J. (1999). Contributions of viral splice sites and cis-regulatory elements to lentivirus vector function. J. Virol. *73*, 6171–6176.
28. Delviks, K.A., Hu, W.S., and Pathak, V.K. (1997). Psi- vectors: murine leukemia virus-based self-inactivating and self-activating retroviral vectors. J. Virol. *71*, 6218–6224.
29. Fang, Y., Gong, X., Xu, M., Zeng, F., and Zhang, J. (2013). A self-deletion lentiviral vector to reduce the risk of replication-competent virus formation. J. Gene Med. *15*, 102–112.
30. Beerens, N., Groot, F., and Berkhout, B. (2001). Initiation of HIV-1 reverse transcription is regulated by a primer activation signal. J. Biol. Chem. *276*, 31247–31256.
31. Beerens, N., and Berkhout, B. (2002). The tRNA primer activation signal in the human immunodeficiency virus type 1 genome is important for initiation and processive elongation of reverse transcription. J. Virol. *76*, 2329–2339.
32. Brodsky, A.S., and Silver, P.A. (2000). Pre-mRNA processing factors are required for nuclear export. RNA *6*, 1737–1749.
33. Perucho, M., Hanahan, D., Lipsich, L., and Wigler, M. (1980). Isolation of the chicken thymidine kinase gene by plasmid rescue. Nature *285*, 207–210.
34. Buckley, S.M.K., Delhove, J.M.K.M., Perocheau, D.P., Karda, R., Rahim, A.A., Howe, S.J., Ward, N.J., Birrell, M.A., Belvisi, M.G., Arbuthnot, P., et al. (2015). In vivo bioimaging with tissue-specific transcription factor activated luciferase reporters. Sci. Rep. *5*, 11842.
35. Butler, S.L., Hansen, M.S.T., and Bushman, F.D. (2001). A quantitative assay for HIV DNA integration in vivo. Nat. Med. *7*, 631–634.
36. Simioni, P., Tormene, D., Tognin, G., Gavasso, S., Bulato, C., Iacobelli, N.P., Finn, J.D., Spiezia, L., Radu, C., and Arruda, V.R. (2009). X-linked thrombophilia with a mutant factor IX (factor IX Padua). N. Engl. J. Med. *361*, 1671–1675.
37. Nathwani, A.C., Davidoff, A., Hanawa, H., Zhou, J.F., Vanin, E.F., and Nienhuis, A.W. (2001). Factors influencing in vivo transduction by recombinant adeno-associated viral vectors expressing the human factor IX cDNA. Blood *97*, 1258–1265.
38. Nathwani, A.C., Davidoff, A.M., Hanawa, H., Hu, Y., Hoffer, F.A., Nikanorov, A., Slaughter, C., Ng, C.Y., Zhou, J., Lozier, J.N., et al. (2002). Sustained high-level expression of human factor IX (hFIX) after liver-targeted delivery of recombinant adeno-associated virus encoding the hFIX gene in rhesus macaques. Blood *100*, 1662–1669.
39. Sakuma, T., Barry, M.A., and Ikeda, Y. (2012). Lentiviral vectors: basic to translational. Biochem. J. *443*, 603–618.
40. Baranyi, L., Doering, C.B., Denning, G., Gautney, R.E., Harris, K.T., Spencer, H.T., Roy, A., Zayed, H., and Dropulic, B. (2013). Rapid generation of stable cell lines expressing high levels of erythropoietin, factor VIII, and an antihuman CD20 antibody using lentiviral vectors. Hum. Gene Ther. Methods *24*, 214–227.
41. Montini, E., Cesana, D., Schmidt, M., Sanvito, F., Bartholomae, C.C., Ranzani, M., Benedicenti, F., Sergi, L.S., Ambrosi, A., Ponzoni, M., et al. (2009). The genotoxic potential of retroviral vectors is strongly modulated by vector design and integration site selection in a mouse model of HSC gene therapy. J. Clin. Invest. *119*, 964–975.
42. Knight, S., Bokhoven, M., Collins, M., and Takeuchi, Y. (2010). Effect of the internal promoter on insertional gene activation by lentiviral vectors with an intact HIV long terminal repeat. J. Virol. *84*, 4856–4859.
43. Wang, Z., Ma, H.-I., Li, J., Sun, L., Zhang, J., and Xiao, X. (2003). Rapid and highly efficient transduction by double-stranded adeno-associated virus vectors in vitro and in vivo. Gene Ther. *10*, 2105–2111.
44. Li, Z., Düllmann, J., Schiedlmeier, B., Schmidt, M., von Kalle, C., Meyer, J., Forster, M., Stocking, C., Wahlers, A., Frank, O., et al. (2002). Murine leukemia induced by retroviral gene marking. Science *296*, 497.
45. Almarza, D., Bussadori, G., Navarro, M., Mavilio, F., Larcher, F., and Murillas, R. (2011). Risk assessment in skin gene therapy: viral-cellular fusion transcripts generated by proviral transcriptional read-through in keratinocytes transduced with self-inactivating lentiviral vectors. Gene Ther. *18*, 674–681.
46. Nash, K.L., and Lever, A.M.L. (2004). Green fluorescent protein: green cells do not always indicate gene expression. Gene Ther. *11*, 882–883.
47. Mock, U., Riecken, K., Berdien, B., Qasim, W., Chan, E., Cathomen, T., and Fehse, B. (2014). Novel lentiviral vectors with mutated reverse transcriptase for mRNA delivery of TALE nucleases. Sci. Rep. *4*, 6409.
48. Graeble, M.A., Churcher, M.J., Lowe, A.D., Gait, M.J., and Karn, J. (1993). Human immunodeficiency virus type 1 transactivator protein, tat, stimulates transcriptional read-through of distal terminator sequences in vitro. Proc. Natl. Acad. Sci. USA *90*, 6184–6188.
49. Liechtenstein, T., Perez-Janices, N., and Escors, D. (2013). Lentiviral vectors for cancer immunotherapy and clinical applications. Cancers (Basel) *5*, 815–837.
50. Finotti, A., Breda, L., Lederer, C.W., Bianchi, N., Zuccato, C., Kleanthous, M., Rivella, S., and Gambari, R. (2015). Recent trends in the gene therapy of β-thalassemia. J. Blood Med. *6*, 69–85.
51. Hoban, M.D., Orkin, S.H., and Bauer, D.E. (2016). Genetic treatment of a molecular disorder: gene therapy approaches to sickle cell disease. Blood *127*, 839–848.
52. Sessa, M., Lorioli, L., Fumagalli, F., Acquati, S., Redaelli, D., Baldoli, C., Canale, S., Lopez, I.D., Morena, F., Calabria, A., et al. (2016). Lentiviral haemopoietic stem-cell gene therapy in early-onset metachromatic leukodystrophy: an ad-hoc analysis of a non-randomised, open-label, phase 1/2 trial. Lancet *388*, 476–487.
53. Barde, I., Laurenti, E., Verp, S., Wiznerowicz, M., Offner, S., Viornery, A., Galy, A., Trumpp, A., and Trono, D. (2011). Lineage- and stage-restricted lentiviral vectors for the gene therapy of chronic granulomatous disease. Gene Ther. *18*, 1087–1097.
54. Ribeil, J.-A., Hacein-Bey-Abina, S., Payen, E., Magnani, A., Semeraro, M., Magrin, E., Caccavelli, L., Neven, B., Bourget, P., El Nemer, W., et al. (2017). Gene therapy in a patient with sickle cell disease. N. Engl. J. Med. *376*, 848–855.
55. Hansson, M.D., Rzeznicka, K., Rosenbäck, M., Hansson, M., and Sirijovski, N. (2008). PCR-mediated deletion of plasmid DNA. Anal. Biochem. *375*, 373–375.
56. Lin, Y.-C., Boone, M., Meuris, L., Lemmens, I., Van Roy, N., Soete, A., Reumers, J., Moisse, M., Plaisance, S., Drmanac, R., et al. (2014). Genome dynamics of the human embryonic kidney 293 lineage in response to cell biology manipulations. Nat. Commun. *5*, 4767.
57. Charrier, S., Ferrand, M., Zerbato, M., Précigout, G., Viornery, A., Bucher-Laurent, S., Benkhelifa-Ziyyat, S., Merten, O.W., Perea, J., and Galy, A. (2011). Quantification of lentiviral vector copy numbers in individual hematopoietic colony-forming cells shows vector dose-dependent effects on the frequency and level of transduction. Gene Ther. *18*, 479–487.
58. Nadai, Y., Eyzaguirre, L.M., Constantine, N.T., Sill, A.M., Cleghorn, F., Blattner, W.A., and Carr, J.K. (2008). Protocol for nearly full-length sequencing of HIV-1 RNA from plasma. PLoS One *3*, e1420.
59. Schambach, A., Bohne, J., Chandra, S., Will, E., Margison, G.P., Williams, D.A., and Baum, C. (2006). Equal potency of gammaretroviral and lentiviral SIN vectors for expression of O6-methylguanine-DNA methyltransferase in hematopoietic cells. Mol. Ther. *13*, 391–400.
60. Bolivar, F., Rodriguez, R.L., Greene, P.J., Betlach, M.C., Heyneker, H.L., Boyer, H.W., Crosa, J.H., and Falkow, S. (1977). Construction and characterization of new cloning vehicles. II. A multipurpose cloning system. Gene *2*, 95–113.
61. Afgan, E., Baker, D., van den Beek, M., Blankenberg, D., Bouvier, D., Čech, M., Chilton, J., Clements, D., Coraor, N., Eberhard, C., et al. (2016). The Galaxy platform for accessible, reproducible and collaborative biomedical analyses: 2016 update. Nucleic Acids Res. *44* (W1), W3–W10.
62. Kim, D., Langmead, B., and Salzberg, S.L. (2015). HISAT: a fast spliced aligner with low memory requirements. Nat. Methods *12*, 357–360.
63. Li, H. (2013). Aligning sequence reads, clone sequences and assembly contigs with BWA-MEM. Genomics *1303*, 1–3.
64. Wang, L., Zoppè, M., Hackeng, T.M., Griffin, J.H., Lee, K.F., and Verma, I.M. (1997). A factor IX-deficient mouse model for hemophilia B gene therapy. Proc. Natl. Acad. Sci. USA *94*, 11563–11566.
65. Kim, J.-Y., Ash, R.T., Ceballos-Diaz, C., Levites, Y., Golde, T.E., Smirnakis, S.M., and Jankowsky, J.L. (2013). Viral transduction of the neonatal brain delivers controllable genetic mosaicism for visualising and manipulating neuronal circuits in vivo. Eur. J. Neurosci. *37*, 1203–1220.
66. Oliphant, T.E. (2007). Python for scientific computing. Comput. Sci. Eng. *9*, 10–20.

YMTHE, Volume *25*

Supplemental Information

Eliminating HIV-1 Packaging Sequences from Lentiviral Vector Proviruses Enhances Safety and Expedites Gene Transfer for Gene Therapy

Conrad A. Vink, John R. Counsell, Dany P. Perocheau, Rajvinder Karda, Suzanne M.K. Buckley, Martijn H. Brugman, Melanie Galla, Axel Schambach, Tristan R. McKay, Simon N. Waddington, and Steven J. Howe

Supplementary Materials

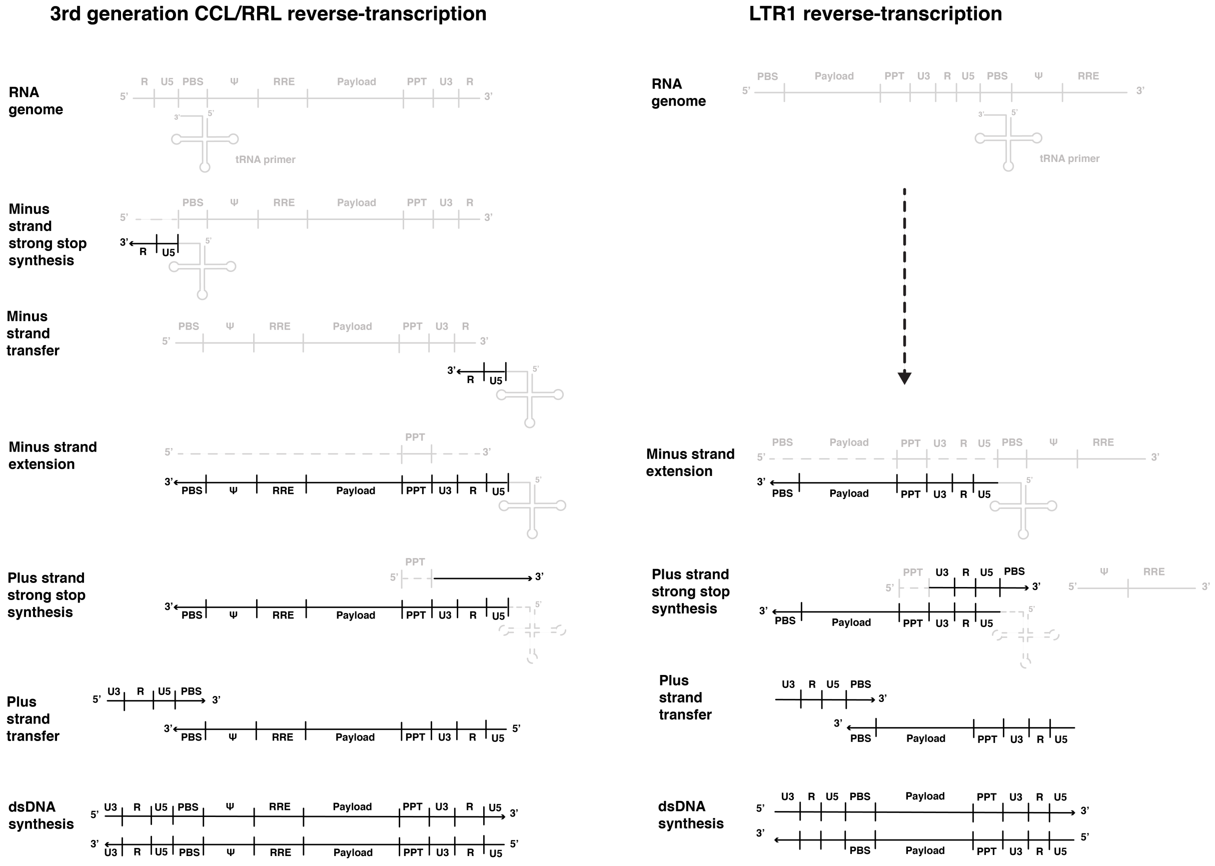

Supplementary Figure S1. LTR1 reverse-transcription requires fewer intermediate steps than a standard lentiviral vector. For all images, grey lines represent RNA, black lines represent the DNA product of reverse-transcription, and dashed grey lines represent RNA degraded by RNAse H. **Left panel.** Conventional lentiviral reverse-transcription can be broken-down in intermediate steps. A tRNA primer binds to the HIV-1 primer-binding site (PBS) on vector RNA and initiates minus strong-stop DNA synthesis, producing single-stranded DNA (ssDNA) complementary to the R-U5 portion of the HIV-1 LTR. Vector RNA is concurrently degraded by RNAse H, thus leaving R-U5 ssDNA free to base pair with the complementary R domain at the 3' end of the vector RNA. Reverse transcriptase proceeds to complete synthesis of minus strand DNA, with PBS at the 5'terminus. Plus strand synthesis is then primed from the polypurine tract (PPT), reading through the 3'LTR (U3-R) and the tRNA, which is complementary to PBS. A single-stranded PBS is now present at both ends, which

permits the second strand-transfer event. Reverse transcriptase then proceeds to complete plus strand synthesis to produce a double-stranded provirus. **Right panel.** LTR1 reverse-transcription operates with fewer intermediate steps than standard lentiviruses. LTR1 vectors lack a 5’LTR, so reverse-transcription is primed on an internal PBS downstream of a solitary HIV-1 LTR (U3-R-U5 domains). This means that reverse-transcriptase can complete minus strand synthesis without performing a strand transfer event and skip straight to minus strand extension. Like conventional reverse-transcription, plus strand synthesis is primed from the PPT, reading through the full-length 3’LTR and tRNA, which is complementary to the PBS. Reading through the tRNA primer displaces the untranscribed RNA at the extreme 3’ end. This process displaces the internal PBS and excludes the HIV-1 packaging sequences from reverse-transcription. As per conventional reverse-transcription, a single-stranded PBS is now present at both DNA termini, which permits the second strand-transfer event and synthesis of a double-stranded DNA provirus. With Ψ-RRE sequences situated downstream of all reverse-transcription events, these sequences are not incorporated into the final LTR1 provirus.

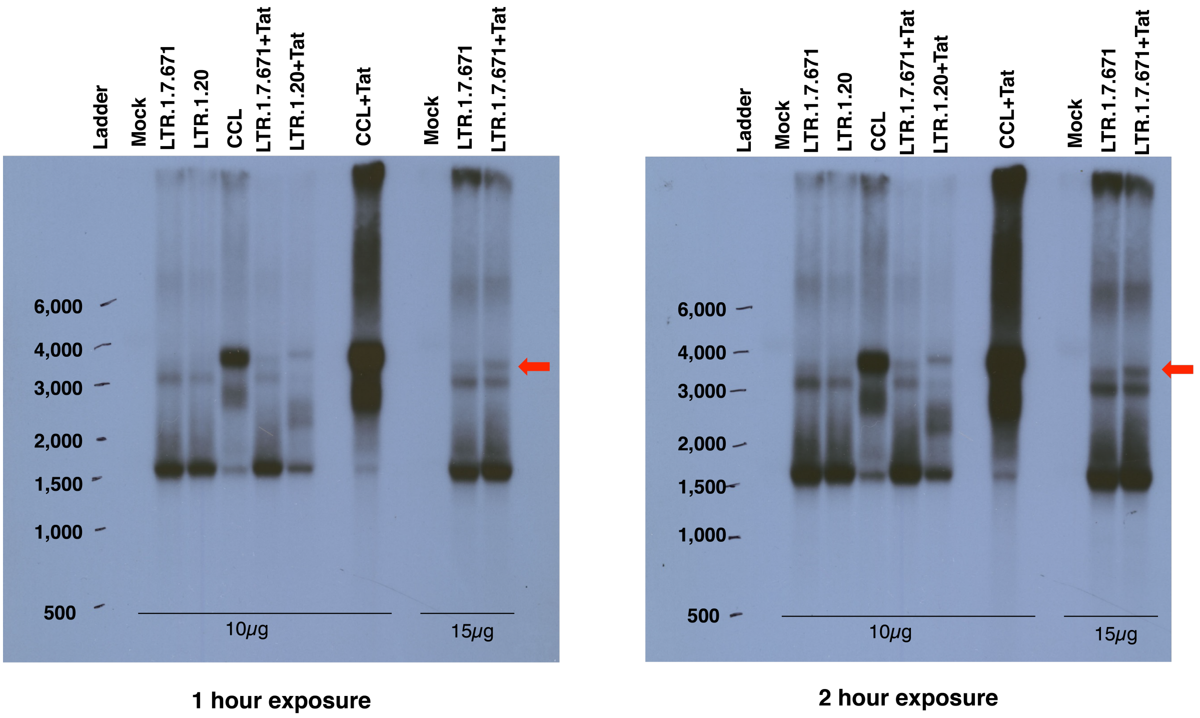

Supplementary Figure S2. Northern blot analysis of LTR1 RNA products. HEK293T cells were transfected with LTR1.7.671-GEW, LTR1.20-GEW or CCL-GEW plasmids. Vectors were produced with or without pcDNA3.Tat during production. RNA was extracted from each treated cell population and processed for northern blot, where RNA was probed for EGFP. EGFP-probed phosphorescence was exposed for 1 hour (left panel) or 2 hours (right panel) at -80°C. Samples were loaded with mass of 10µg, with LTR1.7.671-GEW also loaded at 15µg. Red arrow points to the LTR1.7.671-GEW band assumed to be full-length vector genome transcript. The mock RNA sample was extracted from untreated HEK 293T cells.

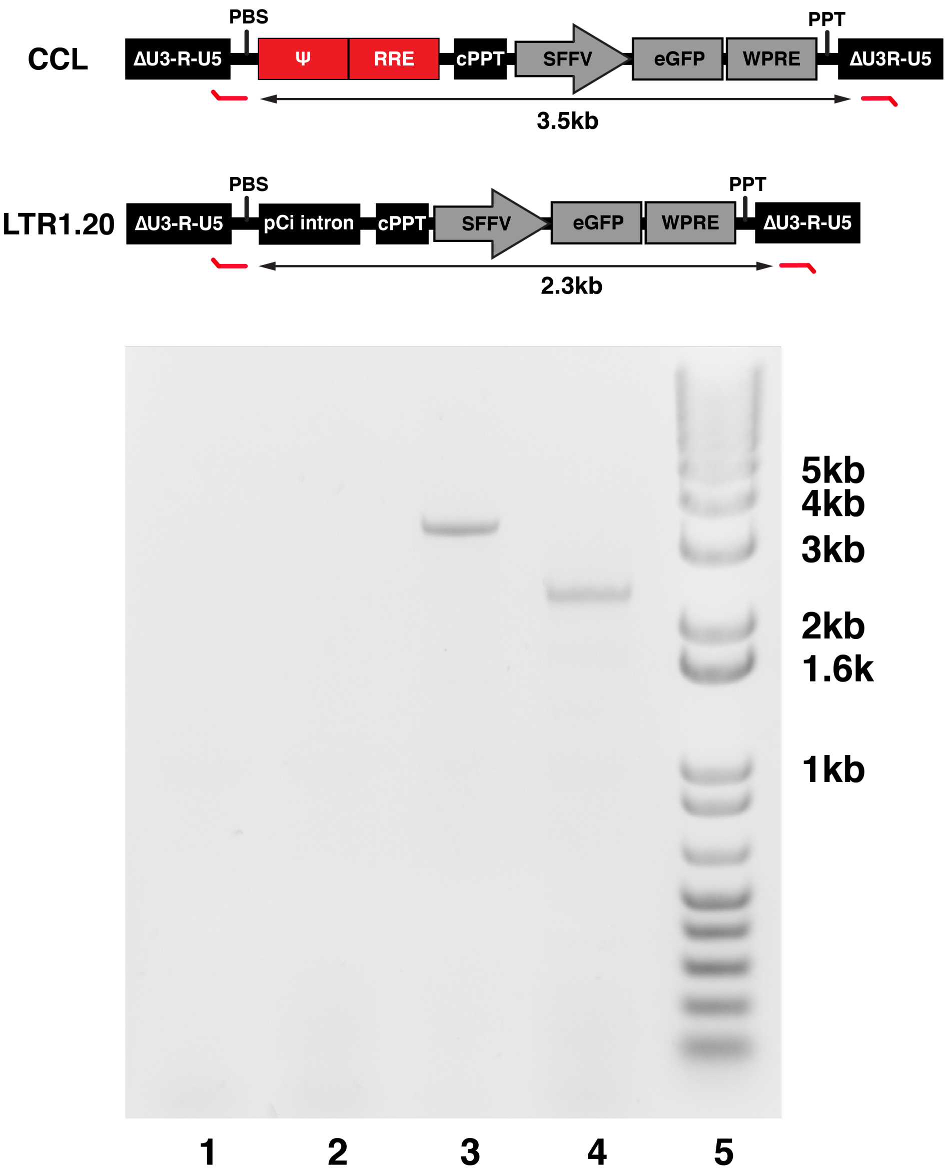

Supplementary Figure S3. PCR amplification of LTR1.20-SFFV-eGFP provirus. Genomic DNA of HT1080 cells transduced with either CCL-SFFV-eGFP (lane 3) or LTR1.20-SFFV-eGFP (lane 4) was amplified using primers directed against the flanking lentiviral LTRs. Water (lane 1) and untransduced HT1080 genomic DNA (lane 2) were devoid of any non-specific amplification. PCR products were analysed alongside the Invitrogen 10kb+ DNA ladder (lane 5), which showed that CCL (3.5kb) and LTR1.20 (2.3kb) samples produced amplicons of the expected sizes. The primer binding sites relative to each provirus have been shown above the gel image.

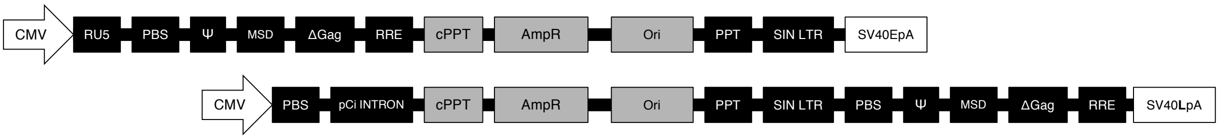

<u>Supplementary Figure S4. Plasmid rescue vector genomes.</u> The pBR322 selection marker, containing an ampicillin resistance gene and bacterial origin of replication, was cloned into the vector transgene region of pCCL (top) or pLTR.20 (bottom). AmpR – ampicillin resistance gene for bacterial selection; Ori – pBR322 plasmid origin of replication for propagation in bacterial cells.

CTTCCCCCTTACCTGACAGCTGTTATTAGATTACGGCCGAATGTCCAATCTTTAACATCATGATGTAGG
GCTCTGTGGTTAACCCATTGCCAAATGTTTTATTATTTCCATAGGTTTAGTGGTCCCAACTTCGGATCTC
ATGTGAGGAAGGTCAAAATGACAGCTTGAAAATATTCCTAACCCGGGGATTGCAGTGCCCATAAGAAAT
GATTGCCAGTGGGGAGAAAGCCCATTTGCTGATTCCACATCTTGTGCTAGCTTTTACACAAATCATCTC
ACAGA**AGAAT**TGGAAGGGCTAATTCACTCCCAACGAAGACAAGATCTGCTTTTTGCTTGTACTGGGTCT
CTCTGGTTAGACCAGATCTGAGCCTGGGAGCTCTCTGGCTAACTAGGGAACCCACTGCTTAAGCCTCA
ATAAAGCTTGCCTTGAGTGCTTCAAGTAGTGTGTGCCCGTCTGTTGTGTGACTCTGGTAACTAGAGATC
CCTCAGACCCTTTTAGTCAGTGTGGAAAATCTCTAGCAGTGGCGCCCGAACAGGGACTGCTAGCCTGG
GCAGGTGTCCACTCCCAGTTCGCTAGCGTTAACTTTTAAAAGAAAAGGGGGGATTGGGGGGTACAGTG
CAGGGGAAAGAATAGTAGACATAATAGCAACAGACATACAAACTAAAGAATTACAAAAACAAATTACAA
AAATTCAAAATTTTATCGATCACGAGACTAGCCTCGAGCCAGGTGGCACTTTTCGGGGAAATGTGCGC
GGAACCCCTATTTGTTTATTTTTCTAAATACATTCAAATATGTATCCGCTCATGAGACAATAACCCTGATA
AATGCTTCAATAATATTGAAAAAGGAAGAGTATGAGTATTCAACATTTCCGTGTCGCCCTTATTCCCTTT
TTTGCGGCATTTTGCCTTCCTGTTTTTGCTCACCCAGAAACGCTGGTGAAAGTAAAAGATGCTGAAGAT
CAGTTGGGTGCACGAGTGGGTTACATCGAACTGGATCTCAACAGCGGTAAGATCCTTGAGAGTTTTCG
CCCCGAAGAACGTTTTCCAATGATGAGCACTTTTAAAGTTCTGCTATGTGGCGCGGTATTATCCCGTAT
TGACGCCGGGCAAGAGCAACTCGGTCGCCGCATACACTATTCTCAGAATGACTTGGTTGAGTACTCAC
CAGTCACAGAAAAGCATCTTACGGATGGCATGACAGTAAGAGAATTATGCAGTGCTGCCATAACCATG
AGTGATAACACTGCGGCCAACTTACTTCTGACAACGATCGGAGGACCGAAGGAGCTAACCGCTTTTTT
GCACAACATGGGGGATCATGTAACTCGCCTTGATCGTTGGGAACCGGAGCTGAATGAAGCCATACCAA
ACGACGAGCGTGACACCACGATGCCTGTAGCAATGGCAACAACGTTGCGCAAACTATTAACTGGCGAA
CTACTTACTCTAGCTTCCCGGCAACAATTAATAGACTGGATGGAGGCGGATAAAGTTGCAGGACCACTT
CTGCGCTCGGCCCTTCCGGCTGGCTGGTTTATTGCTGATAAATCTGGAGCCGGTGAGCGTGGGTCTC
GCGGTATCATTGCAGCACTGGGGCCAGATGGTAAGCCCTCCCGTATCGTAGTTATCTACACGACGGGG
AGTCAGGCAACTATGGATGAACGAAATAGACAGATCGCTGAGATAGGTGCCTCACTGATTAAGCATTG
GTAACTGTCAGACCAAGTTTACTCATATATACTTTAGATTGATTTAAAACTTCATTTTTAATTTAAAAGGA
TCTAGGTGAAGATCCTTTTTGATAATCTCATGACCAAAATCCCTTAACGTGAGTTTTCGTTCCACTGAGC
GTCAGACCCCGTAGAAAAGATCAAAGGATCTTCTTGAGATCCTTTTTTTCTGCGCGTAATCTGCTGCTT
GCAAACAAAAAAACCACCGCTACCAGCGGTGGTTTGTTTGCCGGATCAAGAGCTACCAACTCTTTTTCC
GAAGGTAACTGGCTTCAGCAGAGCGCAGATACCAAATACTGTCCTTCTAGTGTAGCCGTAGTTAGGCC
ACCACTTCAAGAACTCTGTAGCACCGCCTACATACCTCGCTCTGCTAATCCTGTTACCAGTGGCTGCTG
CCAGTGGCGATAAGTCGTGTCTTACCGGGTTGGACTCAAGACGATAGTTACCGGATAAGGCGCAGCG
GTCGGGCTGAACGGGGGGTTCGTGCACACAGCCCAGCTTGGAGCGAACGACCTACACCGAACTGAGA
TACCTACAGCGTGAGCTATGAGAAAGCGCCACGCTTCCCGAAGGGAGAAAGGCGGACAGGTATCCGG
TAAGCGGCAGGGTCGGAACAGGAGAGCGCACGAGGGAGCTTCCAGGGGGAAACGCCTGGTATCTTTA
TAGTCCTGTCGGGTTTCGCCACCTCTGACTTGAGCGTCGATTTTTGTGATGCTCGTCAGGGGGGCGGA
GCCTATGGAAAAACGCCAGCAACGCGGCCTTTTTACGGTTCCTGGCCTTTTGCTGGCCTTTTGCTCACA
TGTGAATTCGAGCTCGGTACCTTTAAGACCAATGACTTACAAGGCAGCTGTAGATCTTAGCCACTTTTT
AAAAGAAAAGGGGGGACTGGAAGGGCTAATTCACTCCCAACGAAGACAAGATCTGCTTTTTGCTTGTA
CTGGGTCTCTCTGGTTAGACCAGATCTGAGCCTGGGAGCTCTCTGGCTAACTAGGGAACCCACTGCTT
AAGCCTCAATAAAGCTTGCCTTGAGTGCTTCAAGTAGTGTGTGCCCGTCTGTTGTGTGACTCTGGTAAC
TAGAGATCCCTCAGACCCTTTTAGTCAGTGTGGAAAATCTCTAGCA**AGAAT**CCTTACACTCTCTGATGC
AGGAGACAATATCCCCAATCACAGGTGAGGGTGCTGACATTAGAGAAGCAAGCCATTCAGCAACAGCT
TAGGGGAAGAGCTCTCAAATGTAGTGTTTTCATACTCCAAAGTTCAGAGGAAGTGTTTCTTTCCCTTAAA
ATGGCAGCAGTTTTCAATTCTGATGTTATAGCTCAGAAGTGGGGACAGAGAGGGATAGTAGAAGGCTG
CCAAATGACATTAAATGAATTTTTTTCATGACAAAGTAATTCCTCAGAATCAGTTTTTTTCCCATTACCTA
ATTGAAGTATCATTATATTCTCATGTTTAATATGTTATTTAGCATATCACCAAGCAGATTGAAAAGGCCG
AAAATGAGCAAAAGAATGCACATATCACTCACTCATTGCCTGTCTTTGCTCTCTCCCTCATGAAGACATA
GAAGAGGAGGCTAGGGGATGCCCCCTCCTCTTCCCTCAGACAGGGGATTCTCACTGACAGAAATATGT
AATTCTTCACATTGCTTTAAAAATGTTCTTCTTCATGTGTATTTATGTGTTTTCAGCTTACTACTCTTATAC
CACCTGCTGTTCAAACAAAAAATCCCACCAAGCCAAGCAGAATTGATGAATATCCATTTACTTTTATTTT
AAAGGGTGCCTCAACTCTTTCAAATCCTAAAATCTTTACCGTATTAATTTGTTAAGGCTTCCTTTAGTAA
GTCAGGGTAAACCCTAA

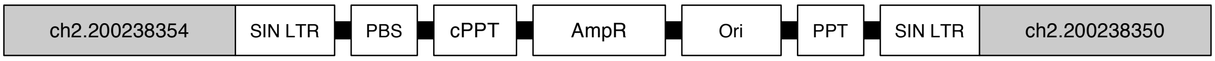

Supplementary Figure S5. Sequence composition of representative plasmid rescue clone. The sequencing data for the rescued LTR1.20 provirus confirms the expected internal provirus structure that would be produced following pCi intron splicing in producer cells (non-shaded text) and shows the repeated dinucleotide pairs at either end of the integrated provirus (bold text) resulting from genomic integration. The shaded text is homologous to human chromosome 2, flanking the repeated dinucleotides at position 200238350-200238354. The map represents the contents and structure of the provirus, devoid of HIV-1 packaging sequences.

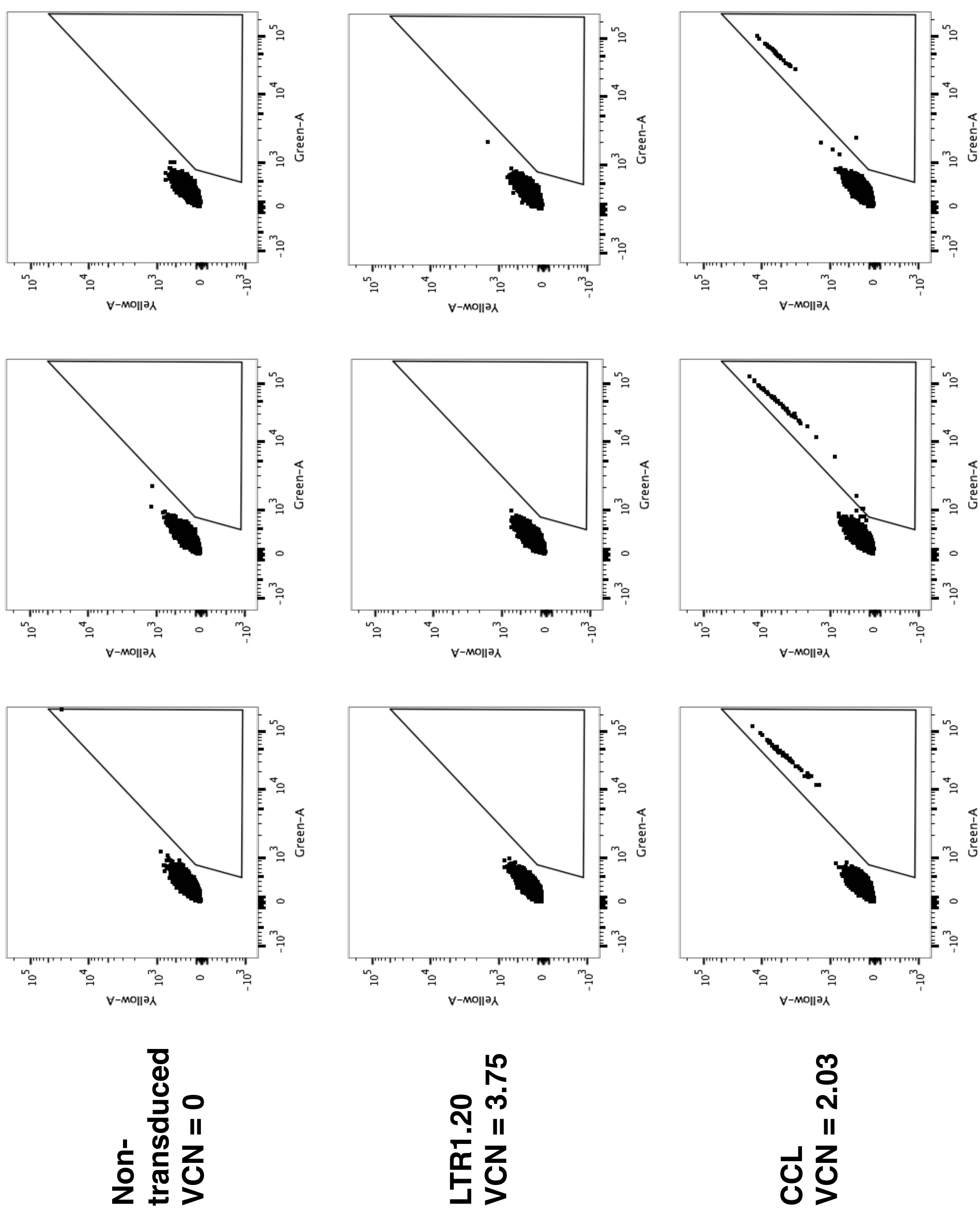

Supplementary Figure S6. Flow cytometry dot plots for remobilisation assay samples. HEK 293T cells transducd with mock remobilised vectors were analysed by flow cytometry for GFP positivity. Vector remobilised by a CCL sample of VCN 2.03 gave clear remobilised GFP vector titres, whilst LTR1.20 showed resistance to remobilisation even a higher VCN dose of 3.75.

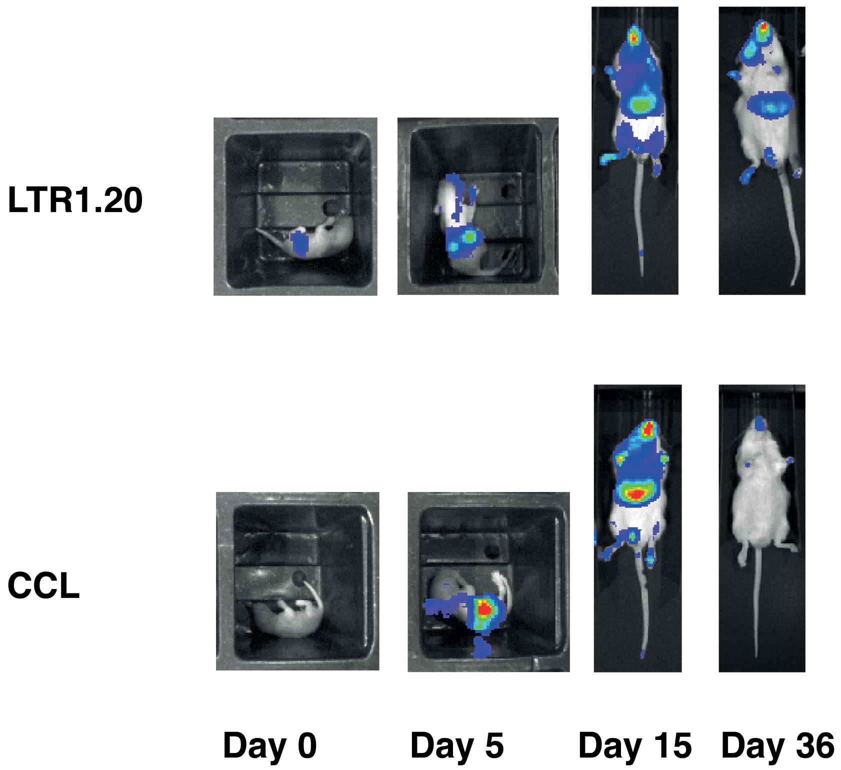

Supplementary Figure S7. Representative bioluminescent images of SFFV-Luc-T2A-eGFP-transduced animals. Outbred CD1 mice received titre-matched intravenous doses of either LTR1.20-SFFV-Luc-T2A-eGFP or CCL-SFFV-Luc-T2A-eGFP at postnatal day 1. Mice were imaged continually throughout the following 36 days to track vector expression *in vivo*. Here, we show representative images taken at 20mins (day 0), 5 days, 15 days and 36 days post-administration.
